# Candida albicans Induces Cross-Kingdom miRNA Trafficking in Human Monocytes To Promote Fungal Growth

**DOI:** 10.1128/mbio.03563-21

**Published:** 2022-02-08

**Authors:** Luke D. Halder, Svitlana Babych, Diana I. Palme, Elham Mansouri-Ghahnavieh, Lia Ivanov, Victory Ashonibare, Daniela Langenhorst, Bhupesh Prusty, Günter Rambach, Melissa Wich, Nora Trinks, Matthew G. Blango, Daniel Kornitzer, Ulrich Terpitz, Cornelia Speth, Berit Jungnickel, Niklas Beyersdorf, Peter F. Zipfel, Axel A. Brakhage, Christine Skerka

**Affiliations:** a Department of Infection Biology, Leibniz Institute for Natural Product Research and Infection Biology, Jena, Germany; b Institute for Virology and Immunobiology, University of Würzburg, Würzburg, Germany; c Division of Hygiene and Medical Microbiology, Medical University of Innsbruck, Innsbruck, Austria; d Department of Cell Biology, Institute of Biochemistry and Biophysics, Friedrich Schiller University, Jena, Germany; e Department of Biotechnology and Biophysics, University of Würzburg, Würzburg, Germany; f Junior Research Group RNA Biology of Fungal Infections, Leibniz Institute for Natural Product Research and Infection Biology, Jena, Germany; g Department of Molecular Microbiology, B. Rappaport Faculty of Medicine, Technion, Haifa, Israel; h Friedrich Schiller University, Jena, Germany; i Department of Molecular and Applied Microbiology, Leibniz Institute for Natural Product Research and Infection Biology, Jena, Germany; University of Texas Health Science Center

**Keywords:** host-pathogen interaction, immune response, monocytes, extracellular vesicles, miRNA, hsa-miR-24-3p, hsa-miR-21-5p, *Candida albicans*, CR3, TLR4, CR1, immune mechanisms

## Abstract

In response to infections, human immune cells release extracellular vesicles (EVs) that carry a situationally adapted cocktail of proteins and nucleic acids, including microRNAs (miRNAs), to coordinate the immune response. In this study, we identified hsa-miR-21-5p and hsa-miR-24-3p as the most common miRNAs in exosomes released by human monocytes in response to the pathogenic fungus Candida albicans. Functional analysis of miRNAs revealed that hsa-miR-24-3p, but not hsa-miR-21-5p, acted across species and kingdoms, entering C. albicans and inducing fungal cell growth by inhibiting translation of the cyclin-dependent kinase inhibitor *Sol1*. Packaging of hsa-miR-24-3p into monocyte exosomes required binding of fungal soluble β-glucan to complement receptor 3 (CR3) and binding of mannan to Toll-like receptor 4 (TLR4), resulting in receptor colocalization. Together, our *in vitro* and *in vivo* findings reveal a novel cross-species evasion mechanism by which C. albicans exploits a human miRNA to promote fungal growth and survival in the host.

## INTRODUCTION

*Candida* spp. are common pathogens in hospital-acquired infections and one of the leading causes of nosocomial bloodstream infections (BSIs) in intensive care unit patients. Recent global estimates found that about 150 million people suffer from serious fungal diseases with an annual incidence of invasive candidiasis of about 750,000, with a high mortality rate (1). About 50% of the global cases of candidemia were reported in Asia, followed by the Americas, Europe, and Africa (1, 2). C. albicans has evolved several evasion mechanisms, including inhibition of the complement system, which allows the fungus to survive in human hosts (3–5). In systemic C. albicans infections, the first step of the immune reaction is activation of immune cells by pattern recognition receptors (PRRs). The most prominent PRRs are the C-type lectin receptors (CLRs), but Toll-like receptors (TLRs) and NOD-like receptors (NLRs) also play important roles in the process of fungal recognition (4). After recognition, an innate immune reaction is initiated primarily in neutrophils, monocytes, and macrophages, ensuring rapid defense against systemic infection. The innate immune response includes complement activation, phagocytosis, generation of reactive oxygen species, proinflammatory cytokine release, and formation of extracellular DNA traps (6–9). In addition, recent work has described extracellular vesicle (EV)-mediated immune reactions to fungal infections (10–13).

EVs are important mediators of signaling in cellular communication, especially among immune cells (14). In 2007, a breakthrough study demonstrated that mRNAs and miRNAs were associated with small EVs called exosomes and that cellular communication could work across species (15). The authors of that report detected mRNA and miRNAs in exosomes harvested from mouse bone marrow-derived mast cells. When exosome-containing mRNA was added to human mast cells, the cells started to produce mouse proteins encoded by the transferred mRNA. This finding opened up a new area of research that highlights the efficiency of EVs in cellular communication (16).

miRNAs are 19- to 24-nucleotide (nt), conserved small RNAs that regulate posttranscriptional gene expression (17). Since their discovery in 1993 (18), more than 2,000 human miRNAs have been annotated; collectively, they are predicted to control 60% of total gene expression (19). However, the miRNAs associated with EVs released from mammalian host cells during fungal infections have not been previously studied. Hence, we sought to characterize the miRNA content of immune cell-derived EVs released during infection and describe the functional activity of hsa-miR-24-3p, one of the most prevalent miRNAs in these vesicles.

## RESULTS

### Monocytes release RNA-containing EVs.

Upon infection with pathogenic fungi, human blood monocytes immediately recognize the microbes and execute a variety of immunological responses, including phagocytosis, release of reactive oxygen species, and formation of extracellular DNA traps. Recently, we reported that upon fungal infection, monocytes release EVs (MEV_Ca_) containing specific cargo and bearing transforming growth factor beta 1 (TGF-β1) on the vesicle surface (11). Since EVs are also known to transport miRNAs that direct the immune response, we were interested in characterizing the miRNAs in MEV_Ca_ released in response to C. albicans. To this end, we first sought to identify the miRNA profile in vesicles and assess the functional consequences of a highly abundant miRNA species. We also aimed to understand how C. albicans induced production of these miRNA-containing EVs.

**FIG 1:**
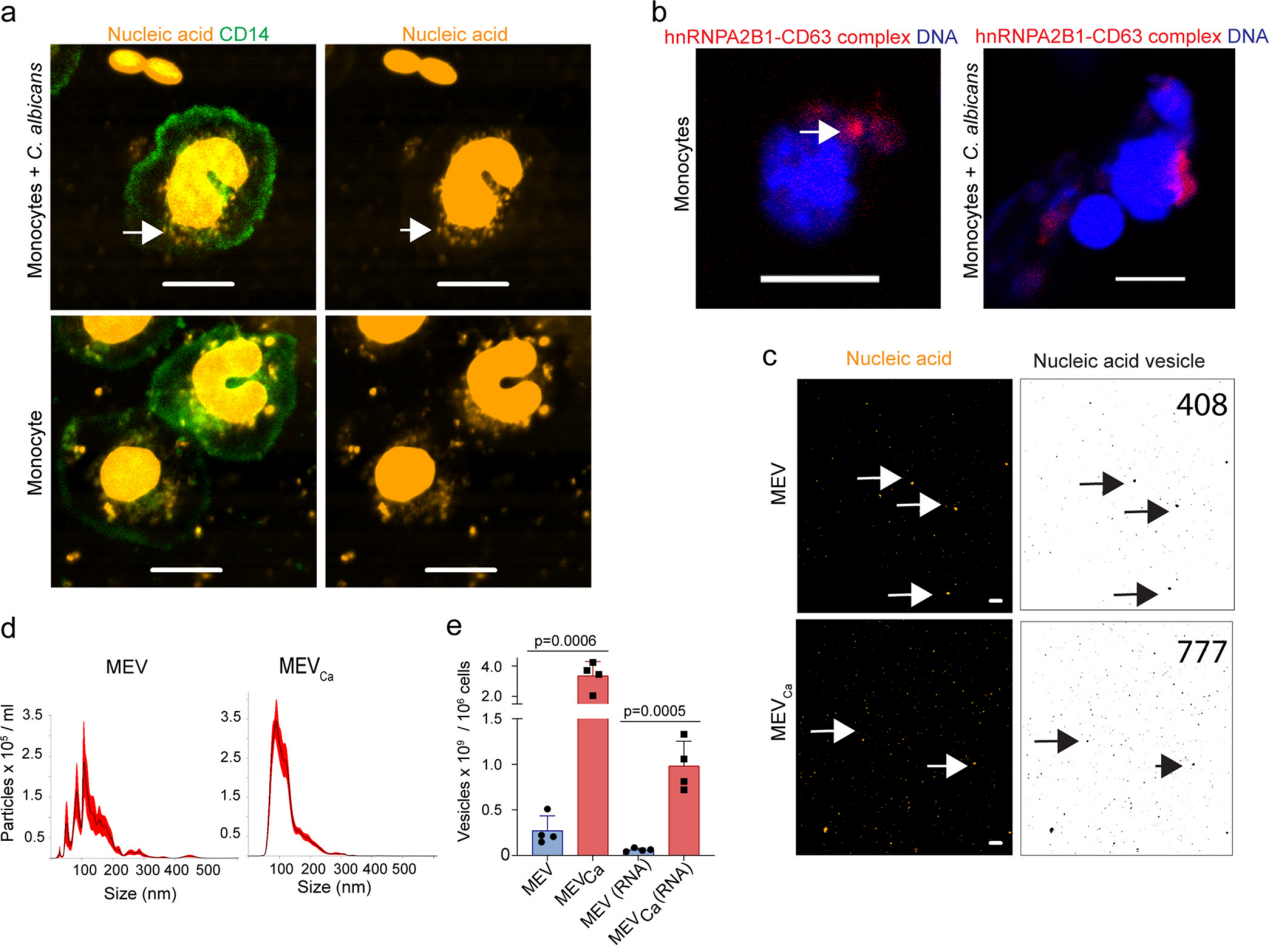
Human monocytes form and release RNA-containing EVs. (a) Vesicles released from human monocytes in response to C. albicans contain nucleic acids as observed by CLSM. Green, CD14; orange, nucleic acids after 1 h of coincubation. Scale bar, 5 μm. Representative data of *n *=* *7 experiments. Green, CD14; orange, nucleic acids. Bars, 10 μm. Uninfected monocytes released fewer vesicles. Data are representative of *n *=* *7 independent experiments. (b) hnRNPA2B1 colocalized with CD63, as shown by proximity ligation assay and CLSM, demonstrating the presence of multivesicular bodies (MVBs) (arrow) in monocytes. Red, hnRNPA2B1/CD63 complexes; blue, DNA. Bars, 10 μm. Data are representative of *n *=* *3 experiments. (c) Isolated vesicles from infected (MEV_Ca_) and uninfected (MEV) monocytes (5 × 10^5^ each cells) contained nucleic acids (arrows). Orange, nucleic acids. Bars, 10 μm. (Right) Nucleic acids containing EVs were counted using ImageJ. (d) Size distribution of MEV and MEV_Ca_, as determined by dynamic light-scattering microscopy (DLSM) using the NanoSight NTA 3.2 software. Graphs were generated by overlaying the size distribution of MEV and MEV_Ca_ derived from *n *=* *4 donors. (e) Total number of MEV_Ca_, as well as RNA-containing MEV_Ca_, increased significantly relative to the number of MEVs and MEVs(RNA). Data represent mean values ± standard deviations (SD), *P = *0.0006 and *P = *0.0005, unpaired two-tailed *t* test, *n *=* *4 different donors. EVs isolated from infected or uninfected monocytes (10^6^ each) were counted by DLSM.

For this purpose, we tracked monocyte-derived vesicles in an *ex vivo* infection model using whole human blood infected with C. albicans. The samples were stained with a monocyte/macrophage-specific anti-CD14 antibody and subjected to live-cell imaging by confocal laser scanning microscopy (CLSM). In the presence of C. albicans, monocytes formed vesicle-like structures around their nuclei as early as 10 min postinfection. These vesicles were also stained with the nucleic acid stain SYTOX orange after permeabilization, indicating that they contained nucleic acids (Fig. 1a; also see Fig. S1a in the supplemental material). Untreated cells released fewer vesicles. Intracellular vesicle formation was independent of phagocytosis of C. albicans and started in the host cytoplasm, suggesting that it took place in multivesicular bodies (MVBs), a hallmark of exosome formation. To ascertain whether the vesicles were in MVBs, we monitored heterogeneous nuclear ribonucleoprotein A2B1 (hnRNPA2B1), which is an RNA sorting protein that binds a specific subset of miRNAs to control their loading into exosomes (20). The protein is primarily localized to the nucleus, and the tetraspanin CD63 is enriched on MVB membranes. We assessed colocalization of hnRNPA2B1 and CD63 in monocytes after C. albicans infection by proximity ligation assay (PLA) and observed colocalization of hnRNPA2B1 and CD63 close to the cell nucleus. Staining also revealed the classical MVB shape, providing further evidence that the exosomes were forming within MVBs (Fig. 1b). Tracking of vesicles 1 h after infection with C. albicans by staining for CD14 and nucleic acids revealed that nucleic acid-containing vesicles accumulated around the cell nucleus as well as extracellularly (Fig. 1a, Fig. S1a and b).

10.1128/mbio.03563-21.1 FIG S1 Characterization of extracellular vesicles. (a and b) Vesicles released from human monocytes in response to C. albicans contain nucleic acids as observed by CLSM. Green, CD14; orange, nucleic acids after 1 h of coincubation. Scale bar, 5 μm. Representative data of *n *=* *7 experiments. Vesicles were detected extracellularly (arrow). (c) Higher concentrations of RNA were isolated from MEV_Ca_ compared to MEV. EVs were isolated from 5 × 10^6^ control or infected monocytes. RNA was measured by NanoDrop (data are presented as mean values ± SD; *P = *0.033, unpaired two-tailed *t* test, *n *=* *3 different donors) (c) and bioanalyzer (d). Particle number of different fractions after size exclusion chromatography detected by DLSM (data are presented as mean values ± SD, *n *=* *3 donors). EVs were isolated from (e) 1 × 10^8^ PBMCs or (f) PBMCs infected with C. albicans (MOI, 1.1). The amount of RNA transporting particles as detected in different fractions of (g) PBMCs or (h) C. albicans infected-PBMC-derived EVs as obtained by size exclusion chromatography measured with DLSM. Download FIG S1, TIF file, 2.7 MB. Copyright © 2022 Halder et al. 2022 Halder et al. https://creativecommons.org/licenses/by/4.0/ This content is distributed under the terms of the Creative Commons Attribution 4.0 International license.

To characterize MEV_Ca_ further, nucleic acids were stained and imaged by CLSM, and stained vesicle spots were counted using ImageJ (Fig. 1c). To determine the RNA content in MEV_Ca_, RNA was isolated from MEV_Ca_ and MEV derived from equal numbers of cells, revealing a significantly higher level of RNA in MEV_Ca_ versus MEV (*P *= 0.033, unpaired two-tailed *t* test, *n *= 3) (Fig. S1c). To characterize the RNA population transported in MEV_Ca_, total vesicle RNA was isolated, resolved by chip-based gel electrophoresis, and analyzed (Fig. S1d). The results revealed the presence of small RNA species in vesicles and confirmed that the RNA content was higher in MEV_Ca_ than in MEV.

To characterize nucleic acids in MEV_Ca_ in more detail, MEV_Ca_ and MEV were isolated by polymer precipitation and examined by dynamic light-scattering microscopy (DLSM) and CLSM. First, MEV_Ca_ size and number were determined based on their Brownian movement in suspension. The number of MEV_Ca_ isolated from C. albicans-infected monocytes (10^6^) was about 10-fold greater than the number isolated from the same number of uninfected cells (Fig. 1d). The size distribution revealed that the vesicles comprised both smaller and larger particles; however, the major vesicle population was smaller than 100 nm, suggesting that exosomes were predominant. To count RNA-transporting vesicles by DLSM, we stained the samples with SYTO RNASelect. RNA-containing vesicles were about 10-fold more abundant in MEV_Ca_ than in MEV (Fig. 1e).

For further detailed characterization, we isolated EVs from C. albicans-infected and untreated peripheral blood mononuclear cells (PBMCs) by size exclusion chromatography and analyzed each fraction by DLSM. In size exclusion chromatography, vesicles were present starting from fraction 5 (Fig. S1e and f). In parallel, fractions were stained with RNA dye, revealing the presence of RNA in fractions 5 to 8 (Fig. S1g and h).

### MEV_Ca_ transport several miRNA species.

**FIG 2:**
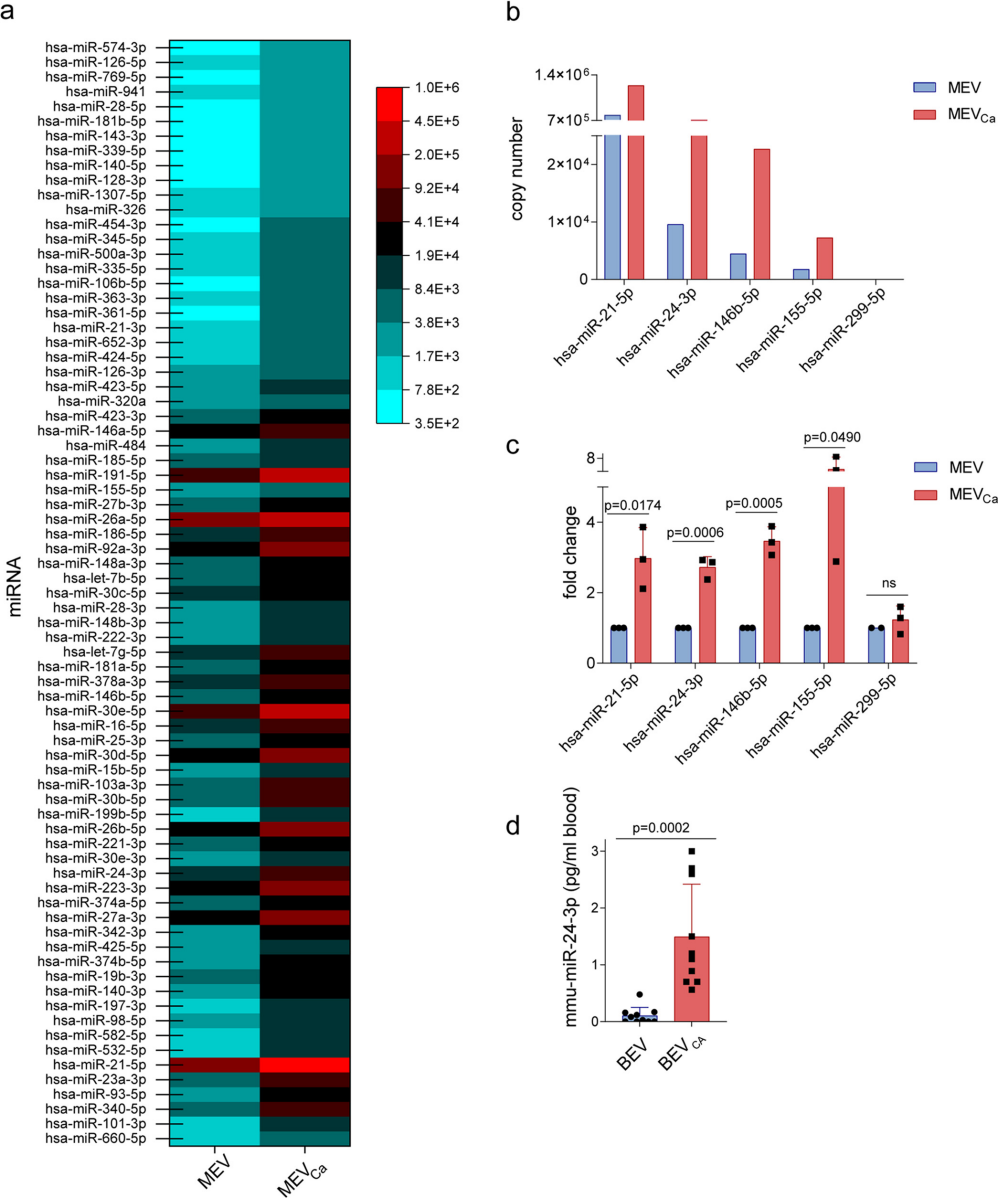
Human monocytes release RNA-associated EVs. RNA isolated from MEV and MEV_Ca_ was analyzed by RNA sequencing using the Illumina HiSeq platform. A total of 75 highly abundant human miRNAs were identified in MEV_Ca_. (a and b) Copy numbers of specific miRNAs were higher in MEV_Ca_ than in MEV. In panels a and b, EV-RNA from eight different donors were pooled before sequencing. RNA was obtained from EVs isolated from infected and uninfected monocytes (each 9 × 10^6^). (c) Selected miRNAs are upregulated in MEV_Ca_ compared with MEV. Upregulation of miRNA was measured by comparative qPCR. Data are presented as means ± SD; unpaired two-tailed *t* test; *n *=* *3 different donors. (d) mmu-miR-24-3p is significantly more abundant in EVs isolated from the blood of C. albicans-infected mice than in those isolated from the blood of uninfected mice. miRNA levels were measured by qPCR. Data are presented as mean values ± SD; *P = *0.0002, unpaired two-tailed *t* test, *n *=* *10 different donors.

The RNA cargo of EVs frequently contains miRNA, which can induce biologically important functional activities in target cells (21). In particular, miRNAs derived from EVs can control the outcome of viral infection (22, 23). To profile the miRNA content associated with MEV_Ca_, we isolated total RNA from MEV_Ca_ and MEV derived from the same number of C. albicans-infected or uninfected monocytes. Vesicle RNA derived from 10 different donors was combined and subjected to deep sequencing (Illumina HiSeq platform). Sequencing revealed 54.7% of the mappable reads as human miRNA. This included 1,381 human miRNAs, of which 75 miRNAs were present in larger amounts in MEV_Ca_ than in MEV (Fig. 2a). hsa-miR-21-5p was the most abundant miRNA, along with the immunomodulatory miRNAs hsa-miR-146b-5p, hsa-miR-155-5p, and hsa-miR-24-3p (Fig. 2b). To validate this result, we isolated RNA from MEV_Ca_ and MEV and subjected the samples to poly(A) tailing, 5′-adapter molecule ligation, and cDNA synthesis. The levels of hsa-miR-21-5p, hsa-miR-24-3p, hsa-miR-146b-5p, and hsa-miR-155-5p were then measured by quantitative PCR (qPCR). The results of this analysis confirmed significant increases in the levels of these miRNAs in MEV_Ca_ versus MEV (*P = *0.0174, *P = *0.0006, *P = *0.0005, and *P = *0.0490 for hsa-miR-21-5p, hsa-miR-24-3p, hsa-miR-146b-5p, and hsa-miR-155-5p, respectively; unpaired two-tailed *t* test, *n *=* *3 different donors), whereas no significant difference was observed for hsa-miR-299-5p (Fig. 2c).

Having shown that hsa-miR-24-3p is associated with isolated vesicles, we then assessed miRNA levels in an *in vivo* infection model. Mice were infected with C. albicans and euthanized 24 h postinfection, and their blood was harvested. Vesicles were isolated from blood of infected (BEV_Ca_) and uninfected (BEV) mice, followed by RNA isolation and qPCR to detect mmu-miR-24-3p, the murine homolog of hsa-miR-24-3p. BEV_Ca_ was associated with significantly more mmu-miR-24-3p (*P = *0.0002; unpaired two-tailed *t* test; *n *=* *10 different donors) than BEV (Fig. 2d), indicating that vesicle-associated mmu-miR-24-3p *in vivo* increased substantially following C. albicans infection.

### hsa-miR-24-3p induces growth of C. albicans.

**FIG 3:**
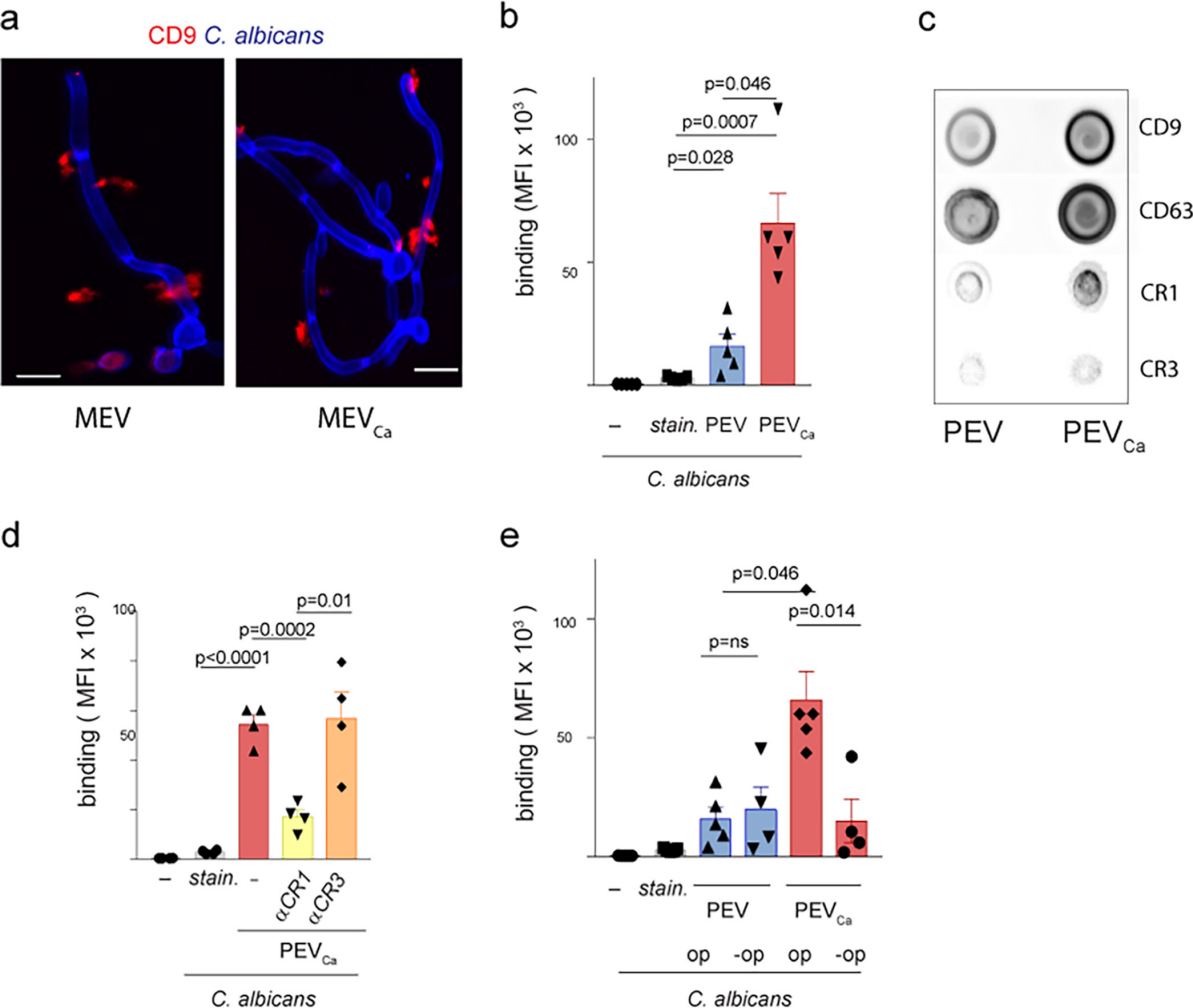
EVs attach to the surface of C. albicans. (a) Human CD9-positive MEV_Ca_ (left) or MEV (right) (each from 5 × 10^5^ monocytes) adhered to the surface of C. albicans, as seen in fixed imaging (CLSM). Blue, C. albicans; red, CD9. Bars, 10 μm. Data are representative of *n *=* *4 independent experiments. (b) C. albicans-induced EVs bound more abundantly than EVs from untreated PBMCs to C. albicans. Stained C. albicans are shown to exclude staining effects. (c) C. albicans-induced EVs from PBMCs exposed CR1, CD9, and CD63 on the surface. (d) Inhibition of CR1 on the EVs substantially reduced the attachment of EVs to C. albicans. (e) EVs from C. albicans-induced PBMCs (PEV_Ca_) are substantially reduced when C. albicans is not opsonized (-op). Data in panels b, d, and e represent mean values ± SD, unpaired two-tailed *t* test; *n *=* *4 independent experiments.

EVs that were released by monocytes were capable of adhering to the surface of C. albicans, as observed by immunofluorescence using antibodies against the EV marker CD9 (Fig. 3a). Approximately three times fewer EVs bound to C. albicans when EVs were isolated from uninfected cells compared to EVs from C. albicans-infected PBMCs (Fig. 3b). This interaction is likely mediated by complement receptor CR1, which is upregulated and surface exposed on the vesicles released by PBMCs upon C. albicans infection (9), as confirmed by dot blot analysis (Fig. 3c). These EVs likely bind to C3b/iC3b deposited (opsonized) by complement activation on the surface of C. albicans (Fig. 3c), as inhibition of CR1 but not CR3 substantially reduced the binding of EVs to C. albicans (Fig. 3d). Similarly, no opsonization resulted in reduced binding of EVs to C. albicans (Fig. 3e).

**FIG 4:**
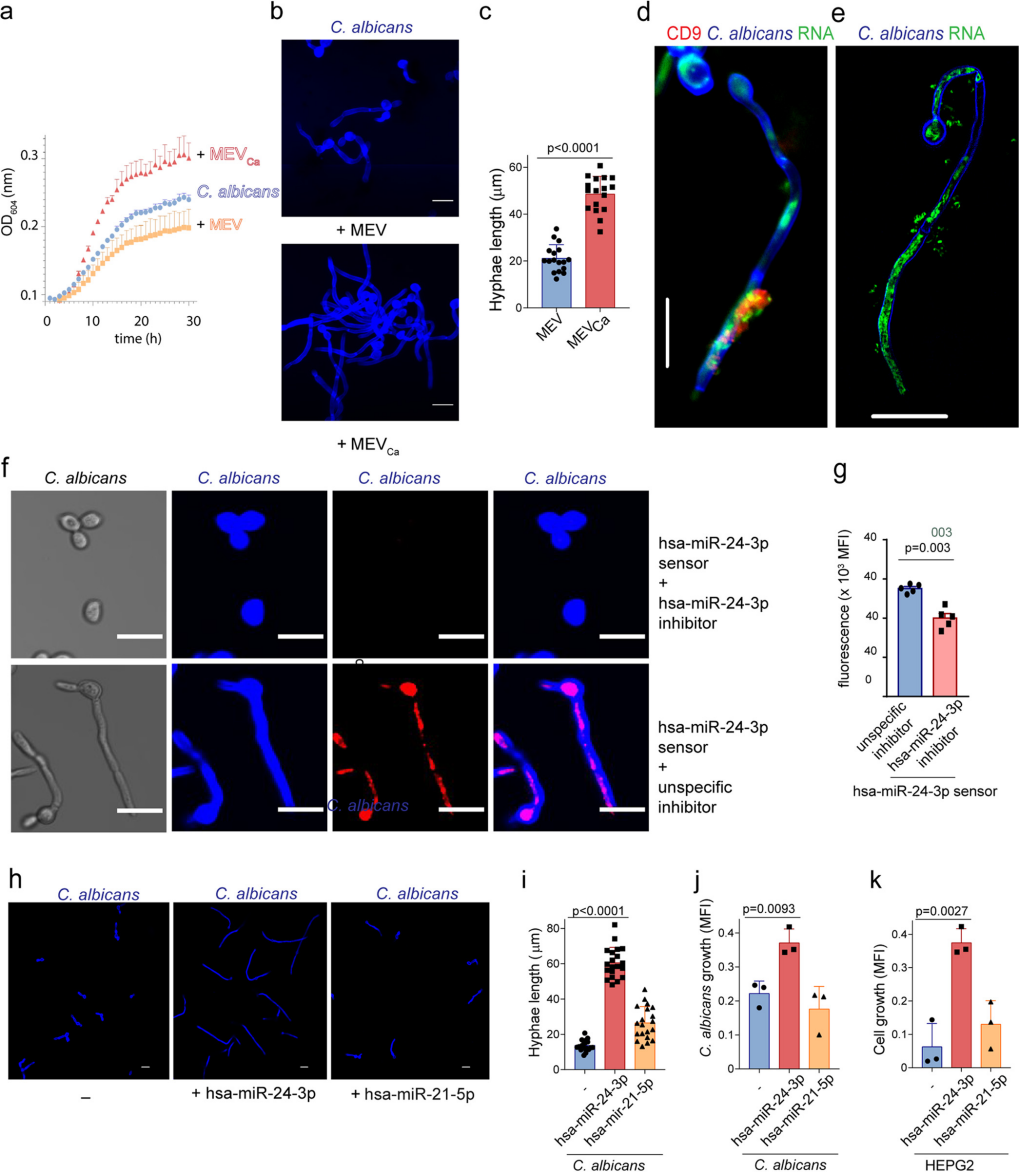
hsa-miR-24-3p increases C. albicans growth. (a) Growth of C. albicans was measured after the fungus was incubated with MEV_Ca_ or MEV. Growth was monitored over 30 h in a plate reader, which measured the OD every hour. Data are presented as mean values ± SD; *n *=* *3 different donors. (b) C. albicans hyphae grew in the presence of MEV_Ca_ but not MEV, as observed by CLSM. Blue, C. albicans. Bars, 10 μm. Data are representative of *n *=* *4 independent experiments. (c) C. albicans hyphae, measured using the ZEN 2011 software, were significantly longer in the presence of MEV_Ca_ than MEV. Data are presented as mean values ± SD, *P* < 0.0001; *n *=* *17; from *n *=* *3 different experiments. (d) RNA from CD9-positive vesicles also migrated into C. albicans, as observed in CLSM. (e) RNA from MEV_Ca_ migrated into C. albicans, as observed by 3D SIM. For panels d and e, data are representative of *n *=* *3 independent experiments. Blue, C. albicans; green, RNA; red, CD9. Bars, 10 μm. (f) hsa-miR-24-3p together with fluorescently labeled hsa-miR-24-3p sensor migrates into C. albicans (lower) but significantly less when hsa-miR24-3p was inhibited in monocytes (upper). (g) Mean values ± SD of fluorescence from 5 independent experiments as measured by a plate reader are shown on the right (*P = *0.003, unpaired two-tailed *t* test). (h and i) Hyphal growth of C. albicans in the presence of hsa-miR-24-3p. Blue, C. albicans. Bars, 10 μm. Data are presented as mean values ± SD; *P* < 0.0001; *n *=* *20; *n *=* *4 different experiments. (j and k) Incubation with hsa-miR-24-3p increased growth of both C. albicans and human hepatic cancer cells (HEPG2), as determined by metabolic activity (CellTiter-Blue assay). Data are presented as mean values ± SD; *P = *0.0093 and *P = *0.0027; unpaired two-tailed *t* test; *n *=* *3 different experiments.

The observation raised the question of whether immune cell-derived vesicles affect fungal growth or other functions. We next monitored C. albicans growth in the presence of MEV_Ca_, since hsa-miR-24-3p was previously shown to promote the growth of certain cancer cells (24). Over 30 h, we monitored C. albicans growth by hourly optical density (OD) measurement. In the presence of MEV_Ca_ (10 h), C. albicans growth was elevated relative to the presence of MEV or no vesicles (Fig. 4a). To confirm acceleration of growth, the fungal cells were grown in the presence of MEV_Ca_ or MEV for 4 h and subsequently fixed and stained, and then the hyphal length was measured in captured images using ZEN 2011 software. In the presence of MEV_Ca_, we observed more rapid C. albicans growth; in addition, hyphal filamentation was 2.5-fold greater (*P* < 0.0001; unpaired two-tailed *t* test; *n *=* *17; three different experiments) (Fig. 4b and c).

Having demonstrated that the vesicles contained miRNAs and attached to the surface of C. albicans cells, we asked whether miRNA was transferred from the vesicle into C. albicans. To this end, we prestained RNA in the MEV_Ca_ with SYTO RNASelect; unbound stain was removed during the vesicle isolation procedure. These stained vesicles were incubated with C. albicans for 4 h, fixed, and subsequently stained for the tetraspanin marker anti-CD9. CD9-labeled EVs adhered to the surface of C. albicans, as demonstrated by CLSM (Fig. 4d). At the same time, labeled RNA from the vesicle migrated from the vesicle into the intracellular portion of the C. albicans hyphae and became distributed within the fungus. This result was confirmed by tracking the RNA using superresolution three-dimensional structured illumination microscopy (3D SIM). This technique suggests that vesicle RNA is delivered into the intracellular lumen of C. albicans and potentially distributed throughout the hyphae (Fig. 4e). To confirm that human miRNA is migrating into C. albicans, human monocytes were incubated with activatable sensor oligonucleotides homologous to hsa-miR-24-3p, as previously described by Yoo et al. (25). Binding of the oligonucleotides to hsa-miR-24-3p resulted in cleavage of the oligonucleotides and subsequent fluorescence of the cleavage products as they became separated from a quencher. The enrichment of fluorescence demonstrated the *in vivo* generation of hsa-miR-24-3p. Vesicles from infected cells treated with sensor oligonucleotides were then incubated with fresh C. albicans, which took up the vesicles containing the miRNA and the sensor oligonucleotides, as seen by fixed imaging of C. albicans (Fig. 4f, lower). However, when monocytes were incubated with LNA (locked nucleic acid) hsa-miR-24-3p inhibitor prior to hsa-miR-24-3p sensors, mature hsa-miR-24-3p was blocked in monocytes for prolonged periods of time, competitively interfering with hsa-miR-24-3p sensors. In this case, C. albicans did not show fluorescence of the sensor oligonucleotides (Fig. 4f, upper, and g). In addition, C. albicans treated with EVs containing hsa-miR-24-3p formed hyphae, while those with hsa-miR-24-3p inhibited just started filamentation (Fig. 4f, Fig. S2). The inhibitors were antisense single-stranded RNA molecules that regulate gene expression by complementary binding and inhibiting mature hsa-miR-24-3p miRNA (25, 26).

10.1128/mbio.03563-21.2 FIG S2 C. albicans filamentation upon vesicle incubation. C. albicans grew longer hyphae upon incubation for 4 h with vesicles from C. albicans induced PBMCs that were previously transfected with hsa-miR-24-3p sensor and an unspecific miR-inhibitor compared to incubation with vesicles from C. albicans induced PBMCs previously transfected with hsa-miR-24-3p sensor plus hsa-miR-24-3p inhibitor. Sensor fluorescence is not shown. C. albicans is stained with calcofluor white. Bar, 10 μm. Representative pictures of 3 independent experiments are shown. Download FIG S2, TIF file, 1.3 MB. Copyright © 2022 Halder et al. 2022 Halder et al. https://creativecommons.org/licenses/by/4.0/ This content is distributed under the terms of the Creative Commons Attribution 4.0 International license.

The experiments confirmed that human miRNA was transferred into C. albicans, where it had the potential to posttranscriptionally regulate gene expression. To determine whether miRNA hsa-miR-24-3p was responsible for promotion of C. albicans growth, we incubated synthetic miRNA hsa-miR-24-3p with C. albicans and monitored growth as before. C. albicans was incubated with hsa-miR-24-3p or hsa-miR-21-5p miRNA for 4 h, fixed, stained with calcofluor white, and observed by CLSM. hsa-miR-24-3p alone increased C. albicans growth and significantly increased hyphal filamentation (3.5-fold, *P* < 0.0001, unpaired two-tailed *t* test, *n *=* *20 from four different experiments) relative to untreated C. albicans (Fig. 4h and i). In contrast to hsa-miR-24-3p, hsa-miR-21-5p had no effect on C. albicans growth. Metabolic activity, which is proportional to the number of C. albicans cells, was measured by CellTiter-Blue assay. hsa-miR-24-3p, but not hsa-miR-21-5p, significantly induced C. albicans growth (*P = *0.0093, unpaired two-tailed *t* test, *n *=* *3 different experiments) within a period of 5 h (Fig. 4j). These observations demonstrated that the human miRNA hsa-miR-24-3p induced growth in C. albicans.

Previous work reported that hsa-miR-24-3p induces cell division by inhibiting cell translation of cyclin-dependent kinase inhibitor (CDKN1B) RNA in mammalian cancer cells (27). Therefore, we incubated human hepatocellular carcinoma cells (HEPG2) with hsa-miR-24-3p, and cell growth was measured by CellTiter-Blue assay. Relative to the untreated control and hsa-miR-21-5p–treated cells, hsa-miR-24-3p–treated cells proliferated more rapidly and showed significant cell growth (*P = *0.0027, unpaired two-tailed *t* test, *n *=* *3 different experiments) (Fig. 4k).

### hsa-miR-24-3p targets *sol1* in C. albicans.

In human cancer cells, hsa-miR-24-3p inhibits protein synthesis of CDKN1B, which in turn inhibits CDKN1/2 protein, an essential factor in cell cycle progression (26, 27). Indeed, inhibition of CDKN1B by hsa-miR-24-3p induces cell division. A functional homolog of CDKN1B in C. albicans, Sic one-like 1 (Sol1), was previously reported to have functions similar to those of cyclin-dependent kinase inhibitor (Sic1) in Saccharomyces cerevisiae (25). Therefore, we hypothesized that hsa-miR-24-3p inhibits translation of s*ol1* mRNA in C. albicans, thereby inducing cell growth. To investigate this hypothesis in detail, we generated a *sol1* reporter C. albicans strain (Sol1-EGFP) by inserting the enhanced green fluorescent protein (EGFP) sequence downstream of the endogenous *sol1* gene.

**FIG 5:**
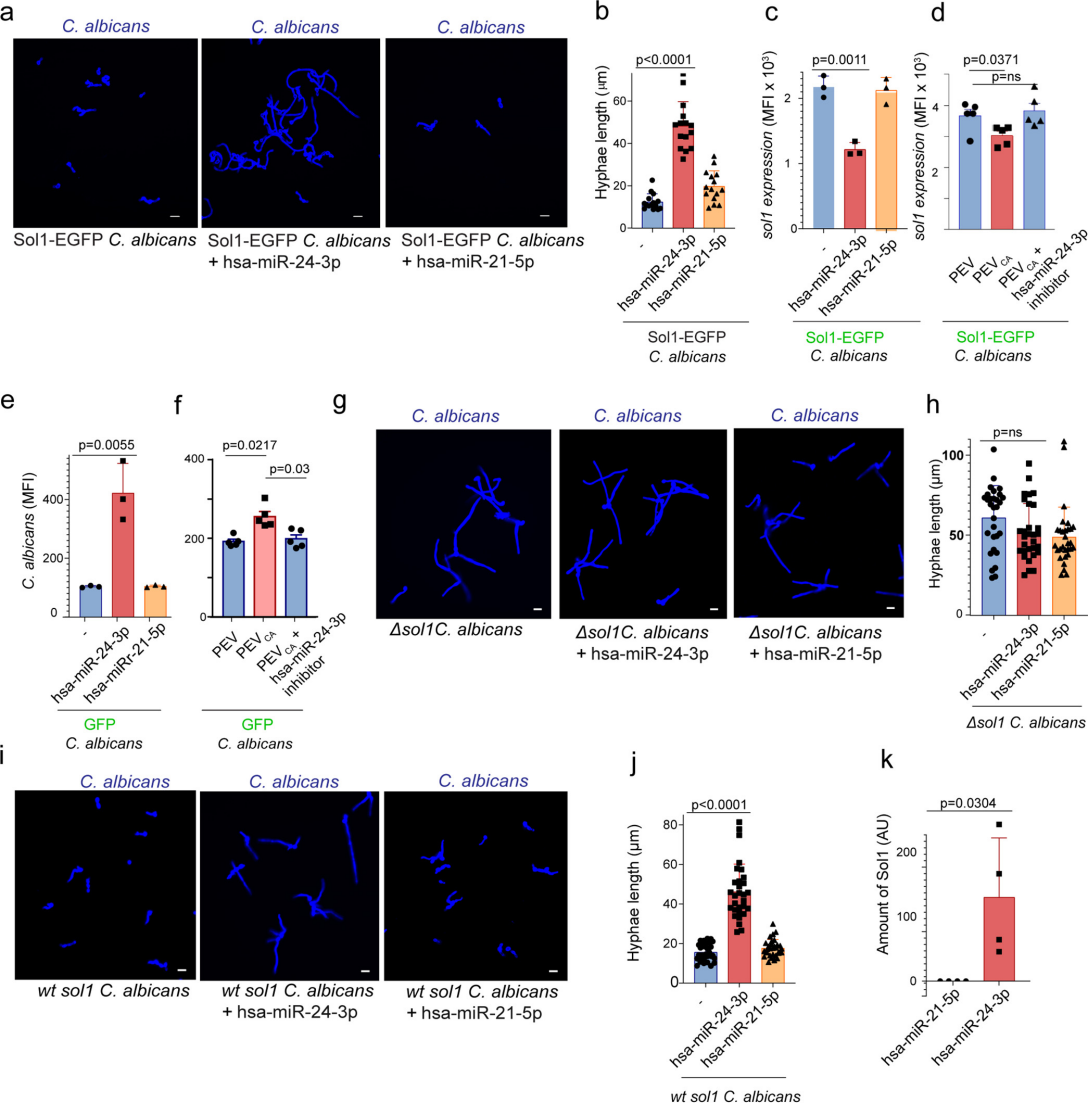
hsa-miR-24-3p inhibits C. albicans *sol1* expression. (a and b) hsa-miR-24-3p increased the growth of Sol1-EGFP-expressing C. albicans, as observed by CLSM after 4 h (a) and hyphal length (b). Blue, calcofluor white-stained C. albicans. Bars, 10 μm. Data represent mean values ± SD, *P* < 0.0001, *n *=* *15, from three different experiments. (c and d) Sol1 expression was reduced in Sol1-EGFP C. albicans when incubated for 18 h with hsa-miR-24-3p (c) or with C. albicans-induced PBMC vesicles (d). Data represent mean values ± SD, unpaired two-tailed *t* test, *P = *0.0011 and *P* = 0.063; *n* = 3 to 5 different experiments in panel c and *n *=* *5 in panel d. (e) C. albicans GFP grew more rapidly in the presence of hsa-miR-24-3p or with C. albicans-induced PBMC vesicles. Data represent mean values ± SD, *P = *0.0055 and *P = *0.0217, unpaired two-tailed *t* test, *n *=* *3 different experiments. Growth was evaluated after 18 h by measuring GFP signals. (g and h) Strong hyphal growth was induced in *Δsol1* C. albicans (KC186) after 4 h but not when the cells were incubated with hsa-miR-24-3p, as observed by CLSM. Blue, C. albicans. Bars, 10 μm. Data represent mean values ± SD, *P* value not significant, *n *=* *30, from three different experiments. (i and j) hsa-miR-24-3p induced significant hyphal growth in *Δsol1* background strain KC643. Blue, C. albicans. Bars, 10 μm. Data represent mean values ± SD, *P* value not significant, *n *=* *30, from three different experiments. (k) Biotinylated hsa-miR-24-3p, but not hsa-miR-21-5p, captured *sol1* mRNA from C. albicans total mRNA, as determined by qPCR. Data represent mean values ± SD, *P = *0.0304, *n *=* *4 different experiments.

We then monitored the growth of Sol1-EGFP C. albicans in the presence of hsa-miR-24-3p by CLSM to confirm that *sol1* is the target gene of hsa-miRNA-24-3p. hsa-miR-24-3p–treated Sol1-EGFP C. albicans grew more rapidly than hsa-miR-21-5p–treated or control cells (Fig. 5a). In addition, hyphal length was greater (∼4-fold) in hsa-miR-24-3p–treated Sol1-EGFP C. albicans (*P* < 0.0001, unpaired two-tailed *t* test, *n *=* *15, from three different experiments) (Fig. 5b). Sol1 inhibition in C. albicans was confirmed 18 h after treatment with hsa-miR-24-3p by monitoring the EGFP signal. Sol1 protein expression was significantly reduced when Sol1-EGFP C. albicans was incubated with synthetic hsa-miR-24-3p (Fig. 5c) or C. albicans-induced PBMC vesicles (PEV_Ca_) (Fig. 5d) but not in fungal cells incubated with synthetic hsa-miR-21-5p or the vesicles from untreated PBMCs (PEV) (*P = *0.0011 and *P = *0.063, respectively; unpaired two-tailed *t* test, *n *= 3 to 5 different experiments). Inhibition of hsa-miR-24-3p with the synthetic hsa-miR-24-3p inhibitor also did not reduce *Sol1* expression (Fig. 5d). Using GFP-expressing C. albicans, we evaluated total GFP fluorescence after treatment with miRNA or PEV_Ca_ for 18 h. hsa-miR-24-3p (Fig. 5e) and PEV_Ca_ (Fig. 5f) significantly increased C. albicans growth, whereas the most abundant miRNA, hsa-miR-21-5p, and PEVs had no effect (*P = *0.0055 and *P = *0.0217, respectively, unpaired two-tailed *t* test, *n *= 3 to 5 different experiments). Using PEV_Ca_ together with the hsa-miR-24 inhibitor did not affect C. albicans growth.

This finding was further validated using Δ*sol1* C. albicans (KC186). As previously reported, Sol1 affects C. albicans morphogenesis (28). Absence of Sol1 resulted in strong filamentation of Δ*sol1* C. albicans (Fig. 5g). In the presence of hsa-miR-24-3p, growth and filamentation were unchanged in Δ*sol1* C. albicans (Fig. 5g and h) (*P* value not significant, *n *=* *30, unpaired two-tailed *t* test from three different donors). In contrast to Δ*sol1* C. albicans, fungal cell growth was elevated in the background strain *sol1* C. albicans (KC643) upon incubation with hsa-miR-24-3p (Fig. 5i and j). These results confirm that human hsa-miR-24-3p regulates fungal *sol1*.

Since this cross-kingdom regulation of a C. albicans gene by a human miRNA was completely unexpected, the findings required further confirmation. Therefore, we pulled down the target mRNA of hsa-miR-24-3p from C. albicans using biotinylated synthetic hsa-miR-24-3p; biotinylated hsa-miR-21-5p was used as a negative control. Biotinylated hsa-miRNA-24-3p or hsa-miRNA-21-5p was allowed to hybridize with C. albicans RNA. The hybridized C. albicans RNAs were captured with streptavidin beads, isolated, and subjected to qPCR with s*ol1* primers. *sol1* amplicon was generated from RNA captured with biotinylated hsa-miR-24-3p but not biotinylated hsa-miR-21-5p (Fig. 5k). Together, these observations demonstrate that C. albicans *sol1* mRNA is targeted by human hsa-miR-24-3p miRNA. Furthermore, a hypothetical binding sequence for hsa-miR-24-3p was identified in the *sol1* gene (Fig. S3).

10.1128/mbio.03563-21.3 FIG S3 Sequence alignment. A potential sequence in the C. albicans *sol1* gene could be targeted by hsa-miR-24-3p. However, this is hypothetical and needs confirmation. Download FIG S3, TIF file, 0.7 MB. Copyright © 2022 Halder et al. 2022 Halder et al. https://creativecommons.org/licenses/by/4.0/ This content is distributed under the terms of the Creative Commons Attribution 4.0 International license.

### hsa-miR-24-3p induces C. albicans growth during infection.

**FIG 6:**
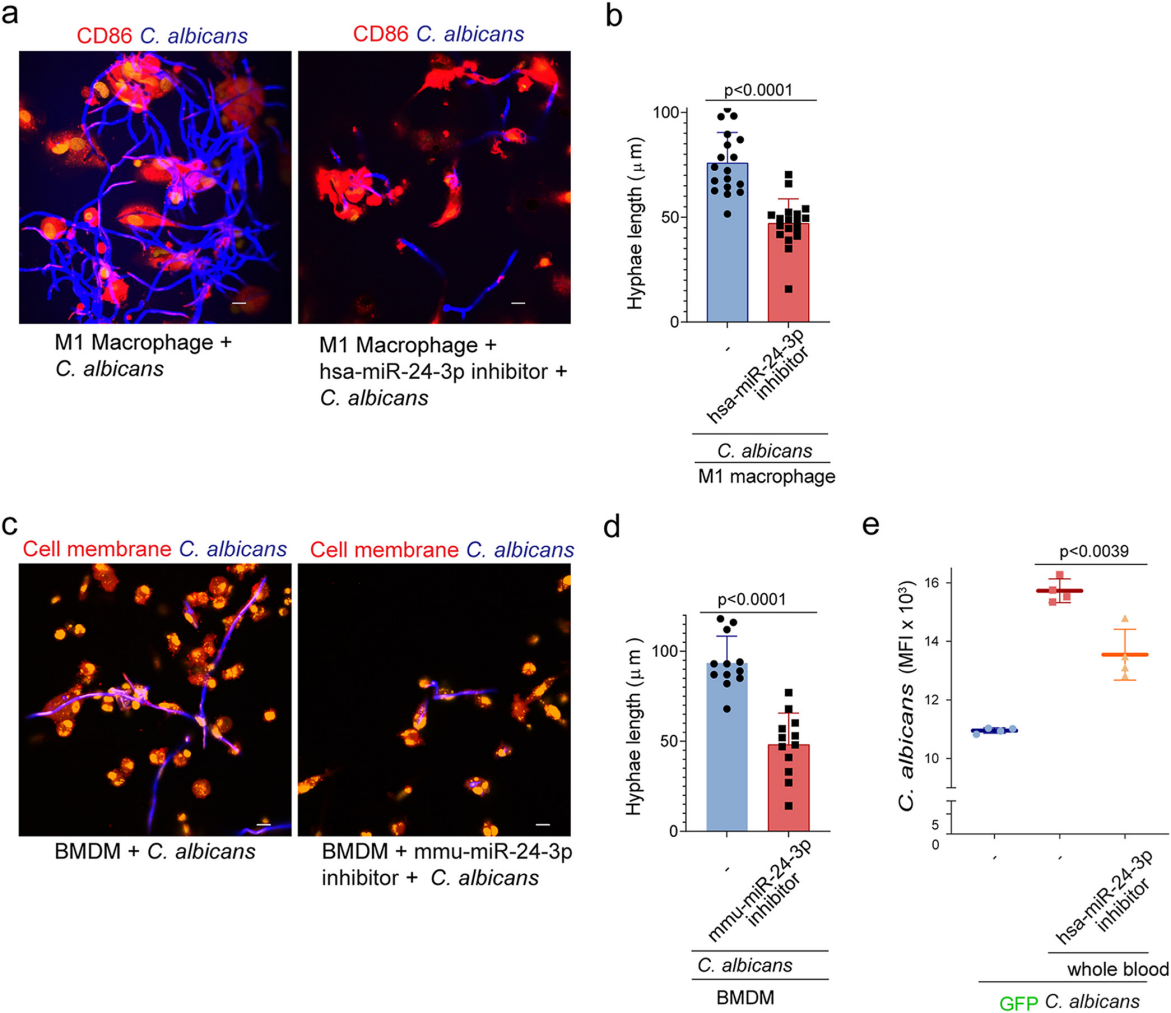
hsa-miR-24-3p induces C. albicans growth during the infection process. (a) When M1 macrophages were infected with C. albicans without any other treatment, C. albicans grew more rapidly than when the M1 macrophages were treated with an inhibitor of hsa-miR-24-3p, as observed by CLSM after 4 h of coincubation. Blue, C. albicans; red, CD86; orange, nucleic acids. Bars, 10 μm. Data are representative of *n *=* *4 independent experiments. (b) hsa-miR-24-3p–inhibited M1 macrophages did not increase hyphal filamentation of C. albicans compared to untreated and infected macrophages, as measured using Zen 2011. Data represent mean values ± SD, *P* < 0.0001, *n *=* *18, from four different experiments. (c and d) Hyphal filamentation of C. albicans was reduced when the cells were incubated with mouse bone marrow-derived monocytes (BMDMo) pretreated with mmu-miR-24-3p inhibitor but not untreated BMDMo, as determined by CLSM. Blue, C. albicans; red, membrane; orange, nucleic acids. Bars, 10 μm. Data represent mean values ± SD, *P* < 0.0001, *n *=* *12 from four different experiments. (e) C. albicans GFP growth was elevated in the presence of whole human blood relative to the untreated control. Inhibition of hsa-miR-24-3p in whole blood significantly decreased C. albicans GFP growth. Growth rates were determined by GFP expression after 18 h. Data represent mean values ± SD, *P = *0.0039, *n *=* *4 different donors.

To determine the physiological relevance of hsa-miR-24-3p miRNA during C. albicans infection, we investigated *in vitro* and *ex vivo* infection models. First, we differentiated M1 macrophages from isolated human blood monocytes and infected them with the yeast form of C. albicans for 4 h. In parallel, we incubated macrophages with the synthetic hsa-miR-24-3p inhibitor. After incubation, the reactions were fixed on glass coverslips; C. albicans was stained with calcofluor white and macrophages with anti-CD86 antibodies against the CD86 surface protein known to be present on these cells. Regular growth of C. albicans with vigorous hyphal filamentation was observed in the presence of untreated control macrophages, but in the presence of macrophages pretreated with hsa-miR-24-3p inhibitor, the growth and filamentation of C. albicans was substantially reduced. Hyphal length measurement revealed significantly reduced hyphal growth in the presence of the hsa-miR-24-3p inhibitor (Fig. 6a and b) (*P* < 0.0001, unpaired two-tailed *t* test, *n *=* *18 from four different experiments). In a similar experiment with mouse bone marrow-derived monocytes, we observed extensive growth of C. albicans with hyphal filamentation in the presence of untreated cells but not in the presence of cells treated with mmu-miR-24-3p inhibitor. Again, C. albicans growth and hyphal filamentation were significantly reduced (*P* < 0.0001, unpaired two-tailed *t* test, *n *=* *12 from four different experiments) (Fig. 6c and d).

To determine whether C. albicans growth could be induced via the cross-kingdom mechanism in an *ex vivo* experiment, we incubated GFP-C. albicans cells with whole human blood and monitored growth by measuring GFP expression after 18 h. As expected, GFP-C. albicans growth was elevated in the presence of whole blood relative to C. albicans alone. When hsa-miR-24-3p in whole blood was inhibited with the synthetic inhibitor before infection with GFP-C. albicans, growth was significantly reduced (*P* = 0.0039, unpaired two-tailed *t* test, *n* = 4 different donors) (Fig. 6e).

### hsa-miR-24-3p–transporting vesicles are released from immune cells due to soluble β-glucan–CR3 and mannan–TLR4 interactions.

**FIG 7:**
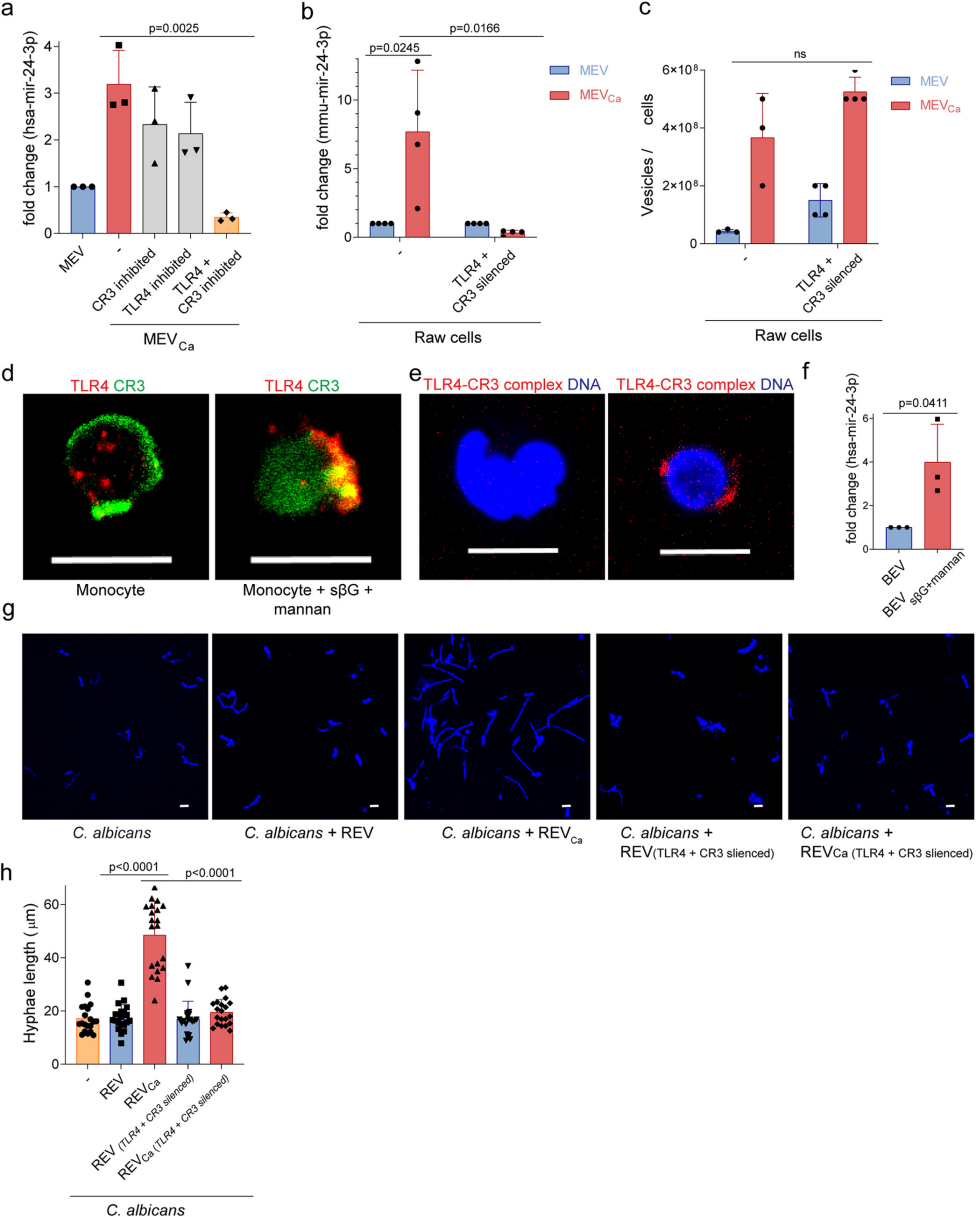
Generation of hsa-miR-24-3p is dependent on CR3 and TLR4. (a) The level of hsa-miR-24-3p 1 h after infection was higher in MEV_Ca_ than in MEV. Blocking of CR3 or TLR4 on blood monocytes had a minor effect on hsa-miR-24-3p content in vesicles. However, when both CR3 and TLR4 were blocked, hsa-miR-24-3p was absent from vesicles. Data represent mean values ± SD, *P = *0.0025, unpaired two-tailed *t* test, *n *=* *3 different donors. EVs were isolated from 10^6^ human monocytes, and hsa-miR-24-3p levels were determined using qPCR. (b) Mmu-miR-24-3p content was significantly higher in REV_Ca_ (vesicles from opsonized C. albicans-induced RAW 264.7 cells) than in REV (vesicles from untreated RAW 264.7 cells). The level of mmu-miR-24-3p was not elevated in *CR3-* or *TLR4*-silenced RAW 264.7 cells. Data represent mean values ± SD, *P = *0.0245 and *P = *0.0166, unpaired two-tailed *t* test, *n *=* *3 or 4 different experiments. (c) *Candida*-treated *CD11b-* and *TLR4*-silenced RAW 264.7 cells generated the same amount of REV as nonsilenced cells. EVs isolated from the same numbers of cells were counted by DLSM. (d) CD11b and TLR4 colocalized on blood monocytes upon incubation with soluble β-glucan (sβG) and mannan but not on untreated cells, as observed by CLSM. Green, CD11b; red, TLR4. Bars, 10 μm. Data are representative of *n *=* *3 independent experiments. (e) Colocalization of CD11b and TLR4 in the presence of soluble β-glucan (sβG) and mannan on monocytes, as confirmed by PLA. Red, CD11b–TLR4 complexes; blue, DNA. Bars, 10 μm. Data are representative of *n *=* *3 experiments. (f) EVs isolated from sβG and mannan-treated human monocytes for 1 h (MEV_sβG+mannan_) contained significantly more hsa-miR-24-3p than MEV. Data represent mean values ± SD, *P = *0.0025, unpaired two-tailed *t* test, *n *=* *3 different donors. REV_Ca_ but not REV_Ca(TLR4 and CD11b silenced)_ induced significant growth (g) and hyphal filamentation (h) in C. albicans. EVs were isolated from opsonized C. albicans-infected or control RAW 264.7 cells (REV and REV_Ca_, respectively). EVs were also isolated from opsonized C. albicans*-*infected or control TLR4- and CD11b-silenced RAW 264.7 cells [REV_(TLR4 and CD11b silenced)_ and REV_Ca(TLR4 and CD11b silenced)_, respectively]. Isolated EVs were counted by DLSM, and C. albicans was incubated with identical amounts of vesicles obtained from the indicated treatments.

The observations described above showed that C. albicans induces human cells to produce miRNA-associated vesicles that promote fungal cell growth and filamentation. Hence, we sought to understand how C. albicans induced production of hsa-miR-24-3p–transporting vesicles. We previously reported that complement receptor 3 (CR3), composed of CD11b/CD18, is important in the release of TGF-β1–transporting vesicles from monocytes. To determine whether CR3 is also required for release of hsa-miR-24-3p-transporting vesicles, we blocked CR3 on monocytes with the anticholesterol drug simvastatin, infected the cells with C. albicans for 1 h, and isolated the vesicles. RNA was extracted from the vesicles and amplified by qPCR to detect hsa-miR-24-3p. The hsa-miR-24-3p content in vesicles derived from CR3-blocked monocytes was reduced but not significantly different from that of vesicles derived from C. albicans-infected monocytes (Fig. 7a). The modest decrease observed from blockage of CR3 led us to explore additional pathogen recognition receptors, like TLR4, which has previously been shown to recognize C. albicans (29). We measured hsa-miR-24-3p content in vesicles after blocking TLR4 on monocytes using a blocking antibody, and again we observed no significant change in release of hsa-miR-24-3p–transporting vesicles. However, when both the CR3 and TLR4 receptors were inhibited in monocytes and the cells were subsequently infected with C. albicans, hsa-miR-24-3p was greatly decreased in EVs (Fig. 7a).

To confirm these results, we knocked down TLR4 and CR3 in murine macrophages (RAW 264.7 cells) using short interfering RNA (siRNA), yielding TLR4- and CR3-silenced cells. Wild-type RAW 264.7 cells upregulated release of mmu-miR-24-3p–transporting vesicles upon C. albicans infection, whereas TLR4- and CR3-silenced RAW 264.7 cells did not exhibit an increase (Fig. 7b). These results confirm that TLR4 and CR3 are required to induce release of mmu-miR-24-3p–transporting vesicles. Notably, similar numbers of vesicles were released from RAW 264.7 cells and TLR4- and CR3-silenced RAW 264.7 cells upon incubation with C. albicans (Fig. 7c). Therefore, we concluded that costimulation of CR3 and TLR4 determines the hsa-miR-24-3p/mmu-miR-24-3p content of EVs but not the total amount of vesicles released by these cells.

To determine which microbial ligand triggers the receptors for release of hsa-miR-24-3p–transporting vesicles, we incubated human monocytes with the CR3 ligand-soluble β-glucan and the TLR4 ligand mannan and then stained CR3 and TLR4 with specific antibodies. Coincubation with mannan and soluble β-glucan led to colocalization of these receptors (Fig. 7d). Colocalization was confirmed by PLA (Fig. 7e). When vesicles were isolated from these coinduced cells, qPCR revealed that their hsa-miR-24-3p content was significantly elevated (Fig. 7f) (*P = *0.0025, unpaired two-tailed *t* test, *n* = 3 different donors).

Having demonstrated that soluble β-glucan and mannan induced TLR4- and CR3-mediated release of hsa-miR-24-3p–transporting vesicles, which in turn induced C. albicans growth, we expected that vesicles generated from cells lacking these particular receptors would not induce C. albicans growth. To test this prediction, we isolated EVs from infected and untreated mouse RAW 264.7 cells and incubated them with C. albicans. Growth was monitored by measurement of hyphal length in fixed-cell imaging. C. albicans exhibited increased growth and significant hyphal filamentation (*P* < 0.0001, unpaired two-tailed *t* test, *n *=* *30, from three different experiments) in the presence of isolated vesicles from C. albicans-infected RAW 264.7 cells but not in the presence of EVs from uninfected cells or EVs from C. albicans-infected and TLR4- and CR3-silenced cells (Fig. 7g and h).

Together, our results reveal a new evasion mechanism by which C. albicans exploits the human vesicle-producing system through binding of β-glucan and mannan to CR3 and TLR4. Furthermore, our data demonstrate a new example of cross-kingdom RNA interference in which a human miRNA is controlled by C. albicans to obtain a growth advantage.

## DISCUSSION

**FIG 8:**
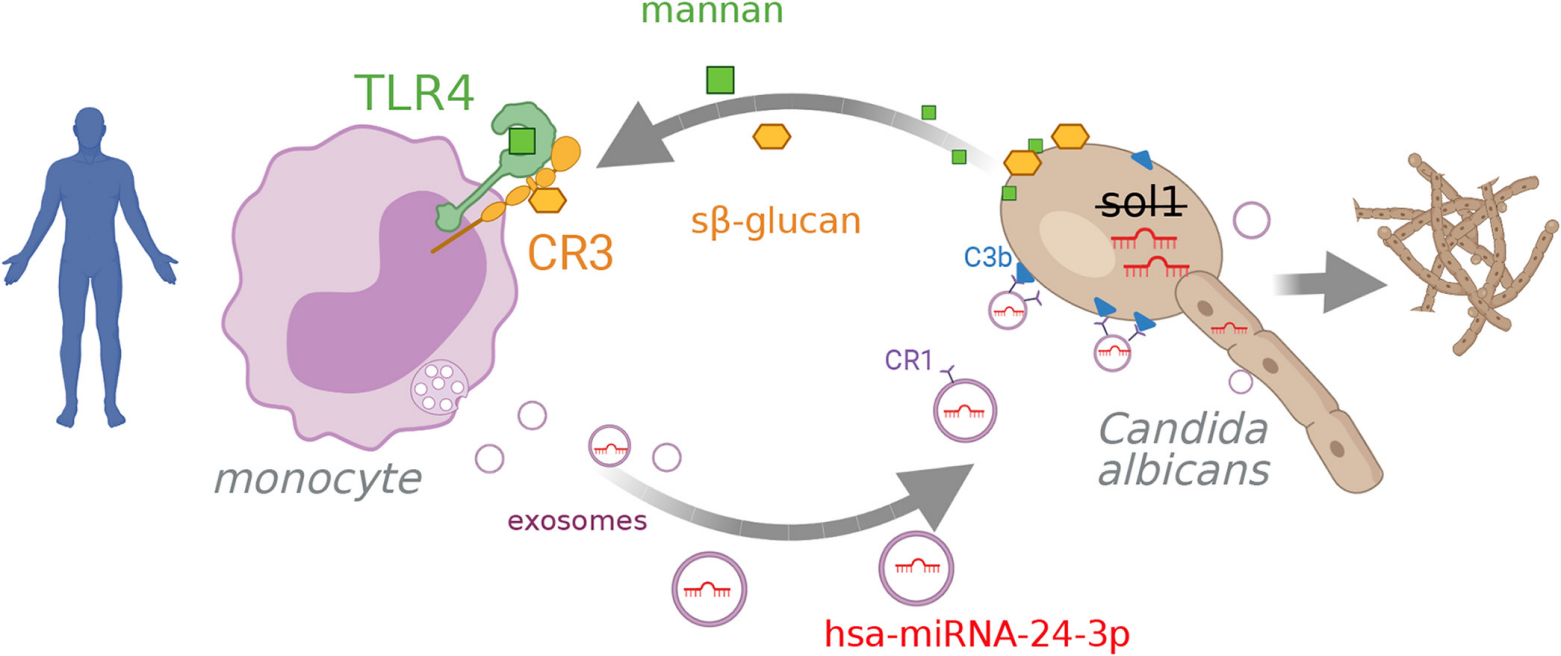
hsa-miRNA-24-3p acts across species boundaries (cross-kingdom) as it regulates C. albicans gene expression. C. albicans induces the release of miRNA-containing vesicles in human monocytes upon binding of mannan and soluble β glucan to TLR4 and CR3, respectively, followed by receptor colocalization. Subsequently, EVs are released from multivesicular bodies transporting miRNAs. EVs attach via CR1 on the vesicle to opsonized C. albicans. hsa-miRNA-24-3p but not hsa-miRNA-21-5p inhibits *sol1* translation in C. albicans, leading to enhanced growth and filamentation of the fungus. Graphic created with BioRender.com.

In this study, we demonstrated that C. albicans exploits the human miRNA signaling system to obtain a growth advantage and evade the host immune response. Over the past decade, studies of EVs have revealed an extraordinary repertoire of functional activities. miRNAs are important constituents of EVs that regulate mRNA posttranscriptionally (30), and this regulation plays a vital role in controlling the outcomes of cancer and viral infections (31, 32). However, the contribution of miRNA has not been extensively studied during bacterial and fungal infections. Our study functionally characterizes an miRNA in immune cell-derived EVs during fungal infection. Our findings show that C. albicans induces production of a vesicle-borne miRNA that acts across kingdoms to promote fungal growth (Fig. 8). This discovery raises new possibilities for fighting fungal infection.

Our study describes vesicle formation and release from human monocytes, confirming previous observations by our group and others (8–10). Previously, immune cell-derived vesicles produced during fungal infection were identified and are of high interest, as these EVs carry immune-modulatory proteins or react against the fungus (10–12). As we confirmed here, C. albicans induced a 10-fold increase in vesicle release from monocytes. Immune cells were already known to spontaneously generate copious amounts of vesicles. However, vesicle contents are known to change under different environmental and disease scenarios, e.g., in cancer progression or viral infection (22, 23).

In this study, the predominant populations of C. albicans-induced EVs were consistent with the known characteristics of exosomes. Initially, EVs were thought to be generated via budding of the plasma membrane. With a size range from 100 to 1,000 nm, they were described as ectosomes, shedding vesicles, microparticles, and microvesicles (MVs) (33, 34). Later, during the early 1980s, a new kind of EV was identified; these particles, which were of endosomal origin and had diameters of 30 to 150 nm, were termed exosomes (35, 36). In this study, CD63 and hnRNP colocalized in C. albicans-infected or control monocytes in MVB-like structures, and the isolated particles were predominantly smaller than 150 nm, which is characteristic of exosomes (35). Together with our previous study, we have shown that the primary EV population produced is exosomes and that hsa-miR-24-3p is transported in small extracellular vesicles. Human cells incubated with C. albicans were stained with specific antibodies (CD63 for vesicles and hnRNPA2B1 for multivesicular bodies) to show that vesicles that are generated in response to C. albicans infection are localized in multivesicular bodies, which are characteristic of exosome formation. Heterogeneous nuclear ribonucleoprotein A2B1 (hnRNPA2B1) is a ubiquitously expressed RNA-binding protein that controls the transport and subcellular localization of miRNAs. hnRNPA2B1 binds a specific subset of miRNAs through their EXOmotifs and controls their loading into exosomes (20). While all of the hnRNPs are present in the nucleus, some shuttle between the nucleus and the cytoplasm. EVs carry a wide range of cargos, including lipids, proteins, and nucleic acids such as dsDNA, mRNA, long noncoding RNAs (lncRNAs), and small RNAs like miRNAs (21, 37). As expected, the vesicles we observed also transported nucleic acids, particularly RNA, and the amount of nucleic acid transporting vesicle increased ∼10-fold upon incubation of monocytes with C. albicans.

Deep sequencing revealed the presence of 75 miRNA species in MEV_Ca_ but significantly fewer in MEV. Several physiologically important miRNAs were identified. In this study, the most abundant miRNA in MEV_Ca_ was hsa-miR-21-5p, which did not affect the growth of C. albicans. However, involvement of hsa-miR-21-5p in inflammation resolution is well documented. hsa-miR-21-5p, induced by LPS in PBMCs via MyD88/NF-κB, is reported to inhibit proinflammatory protein-programmed cell death 4 (PDCD4), ensuring a negative regulation in the TLR4 pathway (38). In macrophages, hsa-miR-21-5p exerts its inflammation regulatory activity by inducing anti-inflammatory cytokine IL-10 and reducing proinflammatory IL-6 (39).

hsa-miR-146a, hsa-miR-146b, and hsa-miR-155, which were abundant in MEV_Ca_, could contribute to controlling the immune response to C. albicans. These miRNAs regulate the immune response to infection and cancer (40). hsa-miR-146a and hsa-miR-146b are important regulators of inflammation generated in response to proinflammatory stimuli; they act in a negative feedback loop that controls excessive inflammation (41). miR-146a and miR-146b inhibit inflammatory transcription mediated by the NF-κB, mitogen-activated protein kinase (MAPK)/EGR, and AP-1 pathway, thereby decreasing production of inflammatory cytokines (41–43). hsa-miR-155 has both pro- and anti-inflammatory functions, depending on the stimulus: overexpression of miR-155 results in polarization of M1 macrophages and induction of proinflammatory signals (44, 45), whereas in mature dendritic cells, miR-155 downregulates proinflammatory cytokine production upon microbial infection (45). Additional study will be required to completely define the full effect of all these miRNA cargo molecules on infection resolution.

This study focused on hsa-miR-24-3p, one of the most abundant miRNAs in MEV_Ca_ released upon C. albicans infection. In contrast to monocytes, the hsa-miR-24-3p increase was not reported in previous work on dendritic cells upon C. albicans infection (46). We also showed that EVs derived from C. albicans*-*infected mouse blood transported substantially more mmu-miR-24-3p, the mouse homolog of human hsa-miR-24-3p, than EVs isolated from uninfected mouse blood. Incubation of C. albicans with isolated MEV_Ca_, but not MEV, induced growth and hyphal filamentation in C. albicans. Surprisingly, RNA from human EVs was taken up by C. albicans. As miRNA species can induce or suppress tumor growth in cancer cells, we hypothesized that human miRNA delivered to C. albicans could contribute to fungal cell cycle regulation and growth. Indeed, hsa-miR-24-3p, but not hsa-miR-21-5p, induced significant C. albicans growth and hyphal filamentation when incubated with the fungus. This observation reveals a so-far-unknown interaction between a host and a pathogenic fungus mediated by exchange of information through EVs. Here, the dependency on hsa-miR-24-3p for enhanced C. albicans growth was demonstrated *in vitro* and *ex vivo* during C. albicans-mediated early infection. We suspect that additional physiologically relevant layers of regulation will be uncovered, building on signaling by EVs. For example, it seems likely that neighboring host cells also detect and respond to the altered C. albicans-induced vesicles produced during infection.

We also sought to understand the process by which hsa-miR-24-3p promotes C. albicans growth. hsa-miR-24-3p, but not hsa-miR-21-5p, also induces growth in human liver cancer cells (HEPG2) (21) and has been reported to promote the growth of cancer cells through inhibition of the cyclin-dependent kinase inhibitor 1B (CDKN1B/p27^kip1^) (24). p27^kip1^ inhibits cyclin-dependent kinases necessary for cell cycle progression and inhibition of p27^kip1^, thereby promoting cell division (47–49). Sic1 in Saccharomyces cerevisiae is a functional homolog of p27^kip1^, and Sol1 in C. albicans is a functional homolog of Sic1 (50). Therefore, we hypothesized that C. albicans s*ol1* mRNA was targeted by hsa-miR-24. Indeed, our experiments confirmed that *sol1* expression was reduced after treatment of C. albicans with hsa-miR-24-3p. Sequence alignment studies revealed a sequence match of hsa-miR-24-3p within the s*ol1* gene (Fig. S3), which needs experimental confirmation. The interaction between human miRNA hsa-miR-24-3p and fungal *sol1* was confirmed by a target pulldown assay followed by *sol1* mRNA identification. This observation suggests that hsa-miR-24-3p released from the human host acts across kingdoms to control growth and filamentation in C. albicans.

To obtain insight into how hsa-miR-24-3p–transporting vesicles are induced in immune cells during C. albicans infection, we investigated two important receptors. We previously reported that fungal soluble β-glucan specifically interacts with the carbohydrate-binding site (LLS domain) of CR3 and that this interaction is required for release of TGF-β1–transporting vesicles (11). Other groups reported that purified mannan from C. albicans induces production of proinflammatory cytokines in human immune cells in a TLR4-dependent manner (29, 51–53). Notably in this regard, the TLR4–NF-κB pathway is responsible for generation of several human miRNAs (54–56). In this study, we found that induction of both CR3 and TLR4 in immune cells by C. albicans-derived soluble β-glucan and mannan induced release of hsa-miR-24-3p–transporting vesicles. These receptors colocalized upon stimulation and were indispensable for functional activity of hsa-miR-24-3p–transporting vesicles.

In summary, we have revealed a novel immune evasion mechanism during fungal infection. Early in infection with C. albicans, blood monocytes release EVs with a large repertoire of functionally important miRNAs, most importantly hsa-miR-24-3p. Soluble β-glucan and mannan from C. albicans induce the generation and release of hsa-miR-24-3p–transporting vesicles by interaction with the CR3 and TLR4 receptors. Surprisingly, these RNA-transporting vesicles attach to the fungal surface via CR1, and hsa-miR-24-3p and likely other RNA species are taken up by C. albicans. EV-mediated transfer of genetic material from human host to pathogenic fungi has not been previously reported. Host-derived hsa-miR-24-3p interacts with C. albicans *sol1* mRNA, thereby downregulating a cell cycle inhibitor, leading to faster C. albicans growth. To date, only a few studies have reported cross-species communication or cross-kingdom transfer of miRNA (16, 57), e.g., in the case of plant-derived miRNAs capable of regulating mammalian receptor pathways (58). Notably, the mechanism we report here acts across kingdoms, emphasizing the striking ability of C. albicans to exploit host signaling pathways. Other miRNA species induced by C. albicans are likely to support C. albicans survival in the host, suggesting that miRNA inhibitors could be used as a therapeutic intervention against candidiasis.

## MATERIALS AND METHODS

### Human cells.

Human monocytes were isolated from sterile buffy coat (Jena University Hospital, Germany) or from fresh blood of healthy donors with informed consent. CPDA and Na-heparin were used as anticoagulants. Using Biocoll density gradient centrifugation (density, 1.077 g/ml) (Biochrom), peripheral blood mononuclear cells (PBMCs) were isolated from blood or buffy coats. Low-speed centrifugation of 160 × *g* was used to remove the platelet population. Using 46% Percoll (density, 1.135 g/ml) (GE Health Care) gradient centrifugation, the lymphocyte population from PBMCs was reduced. Monocytes were then isolated from the lymphocyte-depleted PBMC population by negative selection using the pan-monocyte isolation kit (no. 130-096-537; Miltenyi Biotec) according to the manufacturer’s protocol. Isolated monocytes were verified by the presence of CD14 in flow cytometry.

Human M1 macrophages were generated from lymphocyte-depleted PBMCs. Monocytes from lymphocyte-depleted PBMCs were allowed to adhere to the T75 cell culture flask in M_growth_ medium (RPMI 1640 medium [Lonza] supplemented with 10% fetal calf serum [FCS; ThermoFisher], 2 mM ultraglutamine [Lonza], 25 μg/mL of gentamicin sulfate [Lonza]) for 2 h. M1 macrophages were obtained by differentiation of adherent monocytes in M_growth_ medium supplemented with 50 ng/mL recombinant human granulocyte macrophage colony-stimulating factor (GM-CSF) (Peprotech) for 7 days at 37°C, 95% humidity, and 5% CO_2_. During the differentiation procedure, fresh M_growth_ medium supplemented with GM-CSF was added after 4 days. After 7 days, macrophages were harvested using Accutase (Capricorn Scientific).

The human hepatocellular carcinoma HEPG2 (DSMZ, ACC 180) cells were maintained in M_growth_ medium at 37°C, 95% humidity, and 5% CO_2_.

### Mouse cells.

Mouse experiments were conducted in compliance with European and German regulations. Protocols were approved by the responsible Federal State authority and ethics committee (breeding license 02–052/16 and killing license FSU-02-2017). C57BL/6 mice (Jackson Laboratory) were housed in ventilated cages with free access to water and food. C57BL/6 mice were maintained with heterozygous breeding. Male and female mice with an age between 9 and 22 weeks were used for the experiments. Mice were euthanized with CO_2_. Fresh mouse blood was collected from euthanized mice immediately by cardiac puncture in a hirudin monovette (Sarstedt). Whole blood cells were isolated by centrifugation at 2,000 × *g*, 15 min, 4°C. Serum from the supernatant was used for C. albicans opsonization. Femur and tibia bones were isolated from the euthanized mouse. Bones were sterilized with ethanol, and a bone orifice was cut open and flashed with M_growth_ medium using a 27-gauge needle (B. Braun) to harvest the marrow cells. Collected bone marrow suspension was strained through a 70-μm nylon web. Cells were transferred to ultralow attachment T25 cell culture flasks (Corning). Bone marrow stem cell differentiation was carried out in M_growth_ medium supplemented with 30 ng/mL mouse recombinant M-CSF (BioLegend) at 37°C, 95% humidity, and 5% CO_2_. After 5 days of differentiation, mouse bone marrow-derived monocytes were harvested from the supernatant. The purity of the monocytes was analyzed by detecting the presence of CD115 by flow cytometry using Alexa Fluor 488 anti-mouse CD115 antibody (no. 135512; BioLegend) (1:200).

Mouse RAW 264.7 macrophages (TIB-71; ATCC) were maintained in R_growth_ medium (Dulbecco’s modified Eagle’s medium [DMEM] [Lonza] supplemented with 10% FCS, 2 mM ultraglutamine, 25 μg/mL of gentamicin sulfate) at 37°C, 95% humidity, and 5% CO_2_.

### Microbial strains.

C. albicans wild type (SC5314) (59), GFP-expressing C. albicans (59), a URA3 null mutant of C. albicans, KC643 (CAI4), and s*ol-1* mutant of C. albicans, KC186 (CAI4 *sol1Δ*::*hisG/sol1*Δ::*hisG-URA3-hisG*) (28), were maintained on yeast extract-peptone-dextrose (YPD) agar (2% d-glucose, 1% peptone, 5% yeast extract in agar) for no more than 1 month. After C. albicans was grown overnight in YPD medium at 30°C with shaking, fungal cells were reseeded in fresh YPD medium and grown into the log phase by incubation at 30°C for 4 h prior to the experiments. C. albicans from the log phase was opsonized by incubation in 10% normal human serum at 37°C for 30 min.

SOL1-EGFP C. albicans was generated in the current study using BWP 17 strain (*ura3D*::*imm434*/*ura3D*::*imm434 his1D*/*his1D arg4D*/*arg4D*) (60). EGFP and URA3 coding sequences were inserted downstream of the *sol1* gene using the pGEM-GFP-URA3-GFP plasmid. pGEM-GFP-URA3-GFP was a gift from Bruce Granger (Addgene plasmid number 72606; http://n2t.net/addgene; 72606; RRID:Addgene_72606) (61). The plasmid was predigested with HindIII and ApaI, and PCR was carried out using the following primer (62): forward, 5′-GTAAAGTTAACAAAGAATCAAATGAAAATCAAACCAAAGAGATTATCGTTTGATAATATATCTAAAGGTGAAGAATTATT-3′. The first 60 nucleotides were derived from sequences upstream of the stop codon of C. albicans s*ol1*, while the last 20 nucleotides reflect the sequence directly downstream of the start codon of the EGFP gene sequence in the pGEM-GFP-URA3-GFP plasmid: reverse, 5′-GTGTTTTATCTATTGTCACCTATAAACTATATAAAATCCCTATAATATCAATACTTGACCTCTAGAAGGACCACCTTTGATTG-3′. The first 60 nucleotides were derived from the sequence directly downstream of the stop codon of the C. albicans *sol1* gene, and the last 20 nucleotides reflect the sequence downstream of the stop codon of the URA3 gene sequence in pGEM-GFP-URA3-GFP plasmid. BWP 17 C. albicans was transfected with the purified PCR product and selected for successful transformants using YPD medium in the absence of uracil.

### Vesicle generation and isolation.

Vesicles were isolated from human monocytes, RAW 264.7 cells, and human PBMCs after 1 h of induction or from untreated control cells in either M_induction_ medium (RPMI 1640 medium [Lonza] supplemented with 2 mM ultraglutamine) or R_induction_ medium (DMEM supplemented with 2 mM ultraglutamine) (for RAW 264.7 cells). Cells were washed with Dulbecco’s phosphate-buffered saline (DPBS) twice to remove any serum or FCS. Cells were coincubated with opsonized C. albicans (multiplicity of infection [MOI], 1:1) prior to isolation of infection-derived vesicles. To determine the CD11b and/or TLR4 dependency, monocytes were treated with 30 μM simvastatin (Sigma-Aldrich) and/or 10 μg/mL lipopolysaccharide from Rhodobacter sphaeroides-TLR4 antagonist (InvivoGen) prior to infection with C. albicans. To determine the CD11b and TLR4 dependency in RAW 264.7 cells, CD11b and TLR4 were silenced with Mus musculus CD11b siRNA (no. AM16708; ThermoFisher) and *M. musculus* TLR4 siRNA (no. AM16704; ThermoFisher) (25 pmol/10^6^ cells) by spontaneous transfection for 2 days prior to infection. In the absence of infections, monocytes were also induced with C. albicans-derived sβG (10 μl/10^6^) (9) and C. albicans-derived mannan (200 μg/ml) (WHO International Laboratory of Biological Standard, National Institute for Biological Standard and Control). In all cases, vesicles were isolated from the cell supernatant cleared of cells and cell debris, including apoptotic bodies (63), and C. albicans by centrifugation at 3,000 × *g*, 15 min, at 4°C. EVs from the supernatant were isolated by using ExoQuick-TC (System Biosciences) according to the manufacturer’s protocol.

Vesicles were also isolated from PBMCs and opsonized C. albicans-infected PBMCs using size exclusion chromatography with Sepharose CL-2B (Sigma-Aldrich) according to Böing et al. (64). In total, 15 1-mL fractions were collected from chromatography and subjected to vesicle detection. EVs were used immediately or stored at –20°C. For subsequent use, frozen EVs were thawed slowly on ice.

### Vesicle counting.

Isolated vesicles were counted using NS300 dynamic light-scattering microscopy (Malvern) fitted with NanoSight NTA 3.2 software. Vesicles isolated from 10^6^ monocytes or whole-blood cells were dispersed in 1 mL of DPBS and injected at a 100-AU pump flow rate. Videos were captured at 24 fps, three times at 60 s for each sample, and analyzed using NanoSight NTA 3.2. Fractions of 1 mL obtained from size exclusion chromatography were similarly screened. Isolated vesicles were stained with SYTO RNA Select for counting RNA containing vesicles with the NanoSight software.

### miRNA sequencing.

miRNA sequencing was performed by isolating miRNA from isolated EVs derived from infected (MOI, 1:1) or control human monocytes (each 9 × 10^6^) using the exosomal RNA isolation kit (Norgen Biotek). RNA from 8 different donors was pooled and subjected to deep sequencing using an Illumina HiSeq platform (LC Sciences). Raw sequencing reads (50 nt) were obtained using Illumina’s Sequencing Control Studio software version 2.8 (SCS v2.8) following real-time sequencing image analysis and base-calling by Illumina's Real-Time Analysis, version 1.8.70 (RTA v1.8.70). ACGT101-miR v4.2 (LC Sciences) was used for sequencing data analysis.

### Immunofluorescence.

EV release and C. albicans growth were detected by different immunofluorescence assays. Monocytes were immobilized on poly-l-lysine-coated 12-mm glass coverslips in 24-well plates. Monocytes were infected with opsonized C. albicans (MOI, 1:1) cells for 30 min, 1 h, or 2 h or with 10 μl/10^6^ cells of C. albicans-derived sβG (11) and/or C. albicans-derived mannan (200 μg/ml) (WHO International Laboratory of Biological Standard, National Institute for Biological Standard and Control) for 1 h in M_induction_ medium at 37°C, 95% humidity, and 5% CO_2_. Control monocytes were prepared in a similar manner without infection. After coincubation, cells were fixed with 4% formaldehyde.

For detection of intracellular nucleic acid vesicle, cells were permeabilized with 0.1% saponin and blocked with blocking solution (10% FCS, 1% bovine serum albumin [BSA], and 0.1% Tween 20 in DPBS). CD14 was stained with Alexa Fluor 488 anti-human CD14 antibody (no. 367130; BioLegend) (1:100), CD9 was stained with Alexa Fluor 647 anti-human CD9 antibody (no. MCA469A647T; Bio-Rad) (1:1,000), TLR4 was stained with mouse anti-TLR4 antibody (no. NBP1-51697; R&D Systems) (3 μg/ml) and Alexa Fluor 647 goat anti-mouse IgG (H+L) secondary antibody (no. A-21235; Thermo Fisher) (1:500), and CD11b was stained with rabbit anti-CD11b antibody (no. 133357; Abcam) and Alexa Fluor 488 goat anti-rabbit IgG (H+L) secondary antibody. C. albicans was stained with calcofluor white (Fluka) (1:10), and nucleic acids were stained with SYTOX orange (Thermo Fischer) (5 μM). Coverslips were fixed on a glass slide, and images were captured with an LSM 710 fitted with ZEN 2011 software. 3D SIM microscopy was performed with Zeiss Elyra S.1 SIM (63× oil immersion lens).

Pictures were captured with an LSM 710. Nucleic acids containing vesicles were counted from a captured area of about 212 μm^2^ by subjecting the images to the following Image J (v1.52) algorithm: run(“8-bit”); setAutoThreshold(“Yen dark”);//run(“Threshold…”); setThreshold(40, 255);//setThreshold(40, 255); setOption(“BlackBackground,” false); run(“Convert to Mask”); run(“Watershed”); run(“Analyze Particles…,” “ show=Outlines display clear summarize”); close();

For C. albicans growth observation, extension of hyphae was determined and C. albicans was immobilized on poly-l-lysine-coated 12-mm glass coverslips in a 24-well plate; 2.5 × 10^4^ C. albicans cells were incubated with MEV or MEV_Ca_ isolated from 5 × 10^5^ control and C. albicans-infected (MOI, 1:1) monocytes. C. albicans was also incubated with SYTO RNA select prestained MEV and MEV_Ca_; 2.5 × 10^4^ C. albicans cells were also incubated with 10^8^ REV, REV_Ca_, REV_(TLR4 and CD11b silenced)_, or REV_Ca(TLR4 and CD11b silenced)_ for 4 h in M_induction_ medium at 37°C, 95% humidity, and 5% CO_2_. After coincubation, cells were fixed with 4% formaldehyde and blocked with blocking solution (10% FCS, 1% BSA, and 0.1% Tween 20 in DPBS); 2.5 × 10^4^ C. albicans cells were immobilized with 10 pmol hsa-miR-24-3p or hsa-miR-21-5p for 4 h in M_induction_ medium at 37°C, 95% humidity, and 5% CO_2_. After coincubation, cells were fixed with 4% formaldehyde blocked with blocking solution (10% FCS, 1% BSA, and 0.1% Tween 20 in DPBS). CD9 was stained with Alexa Fluor 647 anti-human CD9 antibody. C. albicans was stained with calcofluor white (Fluka) (1:10). Hypha length was measured with ZEN 2011 software.

For detection of C. albicans growth upon infection, macrophages were immobilized on poly-l-lysine-coated 18-mm glass coverslips in 12-well plates. Macrophages were infected with opsonized C. albicans (MOI, 1:1) for 4 h or incubated with SYTOX RNA select prestained MEV and MEV_Ca_ for 30 min in M_induction_ medium at 37°C, 95% humidity, and 5% CO_2_. After coincubation cells were fixed with 4% formaldehyde. For detection of intracellular nucleic acids, cells were permeabilized with 0.1% saponin and blocked with blocking solution (10% FCS, 1% BSA, and 0.1% Tween 20 in DPBS). CD86 of human macrophage was detected with Alexa Fluor 647 anti-human CD86 antibody (no. 305415; BioLegend). The mouse monocyte membrane was stained with Vybrant DiD cell-labeling solution (ThermoFisher). C. albicans was stained with calcofluor white, and nucleic acid was stained with SYTOX orange (5 μM).

The PLA was used to describe hnRNP1-CD63 complex formation. Monocytes were seeded onto poly-l-lysine-coated 12-mm glass coverslips and infected with opsonized C. albicans (MOI of 1:1) for 30 min in M_induction_ medium at 37°C, 95% humidity, and 5% CO_2_. Cells were fixed with 4% formaldehyde, permeabilized with 0.1% saponin, and blocked with Duolink blocking solution (Sigma-Aldrich). Cells were treated with mouse anti-CD63 (no. NBP2-42225; R&D Systems) (1:500) and rabbit anti-hnRNP1 (no. ab52600; Abcam) antibodies (3 μg/ml). PLA was performed with Duolink in situ red starter kit mouse/rabbit (no. DUO92101; Sigma-Aldrich) according to the manufacturer’s protocol.

The PLA was also used to describe TLR4-CD11b complex formation. Monocytes were seeded onto 6.7-mm poly-l-lysine-coated diagnostic slides. Monocytes were treated with 10 μl/10^6^ cells of C. albicans-derived sβG and (200 μg/ml) C. albicans-derived mannan for 1 h in M_induction_ medium at 37°C, 95% humidity, and 5% CO_2_. Cells were fixed with 4% formaldehyde, permeabilized with 0.1% saponin, and blocked with Duolink blocking solution (Sigma-Aldrich). Cells were treated with mouse anti-TLR4 antibody (no. NBP1-51697; R&D Systems) (3 μg/ml) and rabbit anti-CD11b antibody (no. 133357; Abcam) (3 μg/ml). The PLA was performed with Duolink in situ red starter kit mouse/rabbit (no. DUO92101; Sigma-Aldrich) according to the manufacturer’s protocol.

To track vesicle formation in a whole-blood infection model by live cell imaging, human blood was diluted with M_induction_ medium and incubated with C. albicans cells (MOI, 1:1) in a 30-mm culture dish placed into the incubation chamber of the LSM 710 at 37°C and 5% CO_2_. To observe vesicle formation from monocytes in whole blood, the reaction was treated with Alexa Fluor 488 anti-human CD14 antibody (1:200) and SYTOX orange (5 μM). To observe vesicle generation, pictures were taken every 5 min for 1 h using ZEN 2011.

### CellTiter-Blue assay.

For measuring C. albicans growth by CellTiter-Blue assay, wild-type C. albicans (2.5 × 10^4^) was incubated alone or with 10 pmol synthetic hsa-miR-21-5p or synthetic hsa-miR-24-3p miRNAs in M_induction_ medium in the presence of the CellTiter-Blue reagent (Promega) at 37°C, 95% humidity, and 5% CO_2_. Fluorescence of the cell titer reagent was measured after 5 h according to the manufacturer’s protocol. The CellTiter-Blue assay was also used to determine growth of the cancer cells. HEPG2 cells (5 × 10^4^) were transfected with synthetic hsa-miR-21-5p or synthetic hsa-miR-24-3p miRNAs (each 30 pmol) using Lipofectamine RNAiMAX transfection reagent (ThermoFisher) according to the manufacturer’s protocol. One day after transfection, cells (10^3^) were seeded into 96-well plates in M_induction_ medium at 37°C, 95% humidity, and 5% CO_2_. On day 5, cell growth was measured according to the manufacturer’s protocol of the CellTiter-Blue assay (Promega).

### RNA pulldown assay.

To validate the mRNA target of hsa-miR-24-3p, an assay was performed according to a modified protocol of Torres et al. (65); 60 pmol hsa-miR-24-3p or hsa-miR-21-5p was biotinylated with a Pierce 3′ end biotinylation kit (ThermoFisher) according to the manufacturer’s protocol. C. albicans was cultured overnight at 37°C and 5% CO_2_ in M_induction_ medium. RNA from C. albicans was isolated using a plant/fungus total RNA purification kit (Norgen) by following the manufacturer’s protocol. Biotinylated miRNA was allowed to hybridize with 1 μg RNA from C. albicans in the presence of RNA hybridization buffer (50 mM Tris-HCl, pH 7.0, 750 mM NaCl, 1 mM EDTA, 1% SDS, 15% formamide) for 6 h with agitation. After hybridization, biotinylated miRNA was allowed to immobilize on magnetic streptavidin beads (ThermoFisher) in the presence of 200 U/mL of RNase inhibitor and protease inhibitor cocktail (5 μl/ml) overnight with agitation. Unbound RNA was washed away by magnetic support. miRNA was separated from the magnetic beads by treating with 5 μl proteinase K (20 mg/ml) in 95 μl proteinase K buffer (10 mM Tris-HCl, pH 7.0, 100 mM NaCl, 1 mM EDTA, 0.5% SDS) by incubating at 50°C for 45 min and 95°C for 10 min. RNA was then purified using a total RNA purification kit (Norgen). Purified RNA was subjected to cDNA conversion and qPCR for detection of *sol1* mRNA.

Unspecific miRNA was used as a scrambled control. The hsa-miR-24-3p pulldown for *sol1* mRNA was normalized with the control pulldown to quantify the target mRNA.

### Mouse model of disseminated candidiasis for whole-blood vesicle analysis.

A mouse infection model was used to investigate whether significantly more EVs with mmu-miR-24-3p were also generated upon *in vivo* infection with C. albicans. All experiments were approved by the local Ethics Committee for Animal Care and Use (BMBWF-66.011/0102-V/3b/2018). C57BL/6 mice (Charles River) were housed in ventilated cages with free access to water and food. Male and female mice aged between 9 and 22 weeks were used for the experiments. The mice were infected intravenously with 6 × 10^2^ C. albicans CFU/g body weight. Blood was collected from submandibular vein in EDTA capillary tubes from anesthetized mouse 24 h postinfection. Plasma was isolated by centrifugation at 1,377 × *g* and stored before use at −80°C. Plasma was centrifuged at 3,000 × *g* for 15 min at 4°C to remove cell debris and diluted with M_induction_ medium. EVs were then isolated using ExoQuick-TC (System Biosciences) according to the manufacturer’s protocol. Isolated EVs were stored in −20°C until use. miRNA was isolated from isolated EVs using the exosomal RNA isolation kit (Norgen Biotek), and qPCR was performed to detect mmu-miR-24-3p quantity in the EVs.

### miRNA qPCR.

miRNA was collected from EVs isolated from infected (MOI, 1:1) or control human monocytes (5 × 10^6^) using the exosomal RNA isolation kit according to the manufacturer’s instructions (Norgen Biotek). Polyadenylation and cDNA conversion were performed using MystiCq microRNA cDNA synthesis mix according to the manufacturer’s instructions (Sigma-Aldrich). For qPCR of hsa-miR-21-5p, hsa-miR-24-3p, hsa-miR-146b-5p, hsa-miR-155-5p, and hsa-miR-299-5p, the appropriate specific MystiCq microRNA qPCR assay forward primer (Sigma-Aldrich) was used. In each case, MystiCq microRNA qPCR universal reverse primers were used. Human MystiCq qPCR assay primer SNORD44 (Sigma-Aldrich) was used as a housekeeping control for comparative qPCR of human samples, whereas MystiCq qPCR assay primer SNORD85 (Sigma-Aldrich) was used as a housekeeping control for comparative qPCR of mouse cell samples. To detect mmu-miR-24-3p during *in vivo* infection, quantitative qPCR was performed by obtaining a standard curve for synthetic mmu-miR-24-3p. qPCR was performed in a StepOnePlus real-time PCR system with MystiCq microRNA SYBR green qPCR ReadyMix with ROX (Sigma-Aldrich) with the following parameters: initial denaturation at 95°C for 2 min; 40 cycles of PCR; denaturation at 95°C for 5 s; and annealing, extension, and read (Table 1).

**TABLE 1:** List of primer sequences

Name	Primer sequence
miR-24-3p	UGGCUCAGUUCAGCAGGAACAG
miR-21-5p	UAGCUUAUCAGACUGAUGUUGA
miR-146b-5p	UGAGAACUGAAUUCCAUAGGCU
miR-155-5p	UUAAUGCUAAUCGUGAUAGGGGU
miR-299-5p	UGGUUUACCGUCCCACAUACAU
SNORD44	CCUGGAUGAUGAUAAGCAAAUGCUGACUGAACAUGAAGGUCUUAAUUAGCUCUAACUGACU
SNORD85	UGCAGGGAUGAUACAUACUUGCCCUCACUUAGACCAGAGGUCGAUGAUGAGAGCUUUGUUCUGAGC

### Human cells.

Human monocytes were isolated from sterile buffy coat (Jena University Hospital, Germany) or from fresh blood of healthy donors with informed consent. CPDA and Na-heparin were used as anticoagulants. Using Biocoll density gradient centrifugation (density, 1.077 g/ml) (Biochrom), PBMCs were isolated from blood or buffy coats. Low-speed centrifugation at 160 × *g* was used to remove the platelet population. Using 46% Percoll (density, 1.135 g/ml) (GE Healthcare) gradient centrifugation, the lymphocyte population from PBMCs was reduced. Monocytes were then isolated from lymphocyte-depleted PBMC population by negative selection using the pan-monocyte isolation kit (no. 130-096-537; Miltenyi Biotec) according to the manufacturer’s protocol. Isolated monocytes were verified by the presence of CD14 in flow cytometry.

Human M1 macrophages were generated from lymphocyte-depleted PBMCs. Monocytes from lymphocyte-depleted PBMCs were allowed to adhere to the T75 cell culture flask in M_growth_ medium (RPMI 1640 medium [Lonza] supplemented with 10% FCS [ThermoFisher], 2 mM ultraglutamine [Lonza], 25 μg/mL gentamicin sulfate [Lonza]) for 2 h. M1 macrophages were obtained by differentiation of adherent monocytes in M_growth_ medium supplemented with 50 ng/mL recombinant human GM-CSF (Peprotech) for 7 days at 37°C, 95% humidity, and 5% CO_2_. During the differentiation procedure, fresh M_growth_ medium supplemented with GM-CSF was added after 4 days. After 7 days, macrophages were harvested using Accutase (Capricorn Scientific). Human hepatocellular carcinoma HEPG2 (DSMZ, ACC 180) cells were maintained in M_growth_ medium at 37°C, 95% humidity, and 5% CO_2_.

### Fluorescence *in situ* hybridization.

*In situ* hybridization was performed using a modified protocol (25). The miRNA-24-3p sensor oligonucleotide 5′-[CY5]CUGUUCCUGCUGAACUGAGCCA[BHQ2]-3′ was synthesized by Eurofins. The RNA oligonucleotide was marked at the 5′ end with a Cy5 fluorescent dye and at the 3′ end with black hole quencher 2. miRNA-24-3p miRCURY locked nucleic acid (LNA) miRNA inhibitor and miRCURY LNA miRNA power inhibitor control were purchased from Qiagen (66). Human monocytes (1.2 × 10^6^) were seeded into a 6-well plate and treated for 4 h with anti-miRNA-24-3p LNA antisense oligonucleotides or negative-control inhibitor using HiPerFect transfection reagent (Qiagen) in M_growth_ medium at 37°C, 95% humidity, and 5% CO_2_ (at 5-fold excess of antisense oligonucleotide to sensor). Monocytes pretreated with antisense oligonucleotide inhibitor or with a negative-control inhibitor were incubated with sensor oligonucleotides (1 pmol/μl) and incubated for 4 h. To induce vesicle generation and release, the monocytes were washed three times with 1× PBS and incubated with opsonized C. albicans (MOI, 10) for 2 h in M_induction_ medium at 37°C, 95% humidity, and 5% CO_2_. Subsequently the supernatant was centrifuged at 3,000 × *g* for 15 min.

To assess vesicle uptake by C. albicans, fresh C. albicans cells were incubated with the supernatant separated by a 1-μm-pore-size membrane in a transwell chamber (Corning). After 0.5 h, an orbital shaker was used for 3.5 h at 37°C, 95% humidity, and 5% CO_2_. After coincubation, C. albicans cells were washed (1× PBS), fixed in 4% formaldehyde, permeabilized with 0.1% saponin, and blocked with blocking solution. C. albicans was stained with calcofluor white and the fluorescence signal was recorded using the plate reader Safire2 (Tecan) and by laser scanning microscopy (LSM 710).

### Binding assay.

Vesicle binding to C. albicans was analyzed using a modified protocol by Zhao et al. (67). Extracellular vesicles from PBMCs (1 × 10^6^) were stained with Vybrant DiD cell-labeling solution (2 μM) (Thermo Fisher Scientific) for 5 min at room temperature (RT). For blocking assays, EVs were incubated with monoclonal CR1 or CR3 antibody (each 5 μg/ml) (BioLegend) in exosome-depleted fetal bovine serum (FBS) (1%, diluted with 1× PBS) for 30 min at room temperature. The labeling or blocking reaction was stopped with exosome-depleted FBS (System Biosciences) (1% diluted with 1× PBS) and vesicles isolated using the total exosome isolation solution (Thermo Fisher Scientific) and centrifugation at 10,000 × *g* for 1 h. The washing step was repeated twice and the EV pellet resuspended in exosome-depleted FBS (1% diluted with 1× PBS). Opsonized C. albicans cells (5 × 10^5^) were incubated with EVs for 30 min with shaking at 37°C. In parallel, untreated C. albicans cells were stained with Vybrant DiD cell-labeling solution (2 μM). Yeast cells were washed three times with 1× PBS and binding of vesicles was recorded by flow cytometry using BD Accuri C6 plus (Becton Dickinson).

### CR1 expression on vesicles.

Dot blot assays were used to identify proteins transported by EVs. Isolated EVs were lysed using radioimmunoprecipitation assay lysis buffer (Santa Cruz Biotechnology). EV lysates (4 μl) were spotted onto nitrocellulose blotting membrane and dried at RT for 10 min. Blocking of the membrane was performed in TBS-T buffer (2.4 g Tris base, 8.8 g NaCl, 1 mL Tween 20 [Sigma-Aldrich] diluted in 1 liter of distilled water [pH 7.6], added milk powder [3%], BSA [1%], Tween 20 [0.1%]) and was mixed for 1 h at RT using the rotating roller mixer SRT1 (Stuart Scientific). Afterwards the membrane was incubated with monoclonal antibody (MAb) CD11b (BioLegend), MAb CD35 (BioLegend), MAb CD9 (Thermo Fisher Scientific), and MAb CD63 (Merck) in blocking buffer overnight at 4°C using the rotating roller mixer. The membrane was then washed 3 times in TBS-T buffer for 10 min and incubated with the horseradish peroxidase-conjugated secondary antibody (Dako) at RT for 1 h using the rotating roller mixer SRT1. The membrane was washed 3 times in TBS-T buffer for 10 min each and subsequently incubated with an enhanced chemiluminescence (ECL) mix for 30 s. ECL mix was combined of solution A (luminol and enhancer solution) and B (peroxide solution) from Cheluminate-HRP PicoDetect (AppliChem). Signals from the nitrocellulose blotting membrane were assayed in Fusion FX imaging system (Vilber).

### Data availability.

The sequencing data are accessible at the Gene Expression Omnibus under accession no. GSE186234.
