# Supplementary figures and images for: Candida albicans Induces Cross-Kingdom miRNA Trafficking in Human Monocytes To Promote Fungal Growth
Source: mBio. 2022 Feb 8;13(1):e03563-21. doi: 10.1128/mbio.03563-21 (PMC8822622; doi:10.1128/mbio.03563-21)

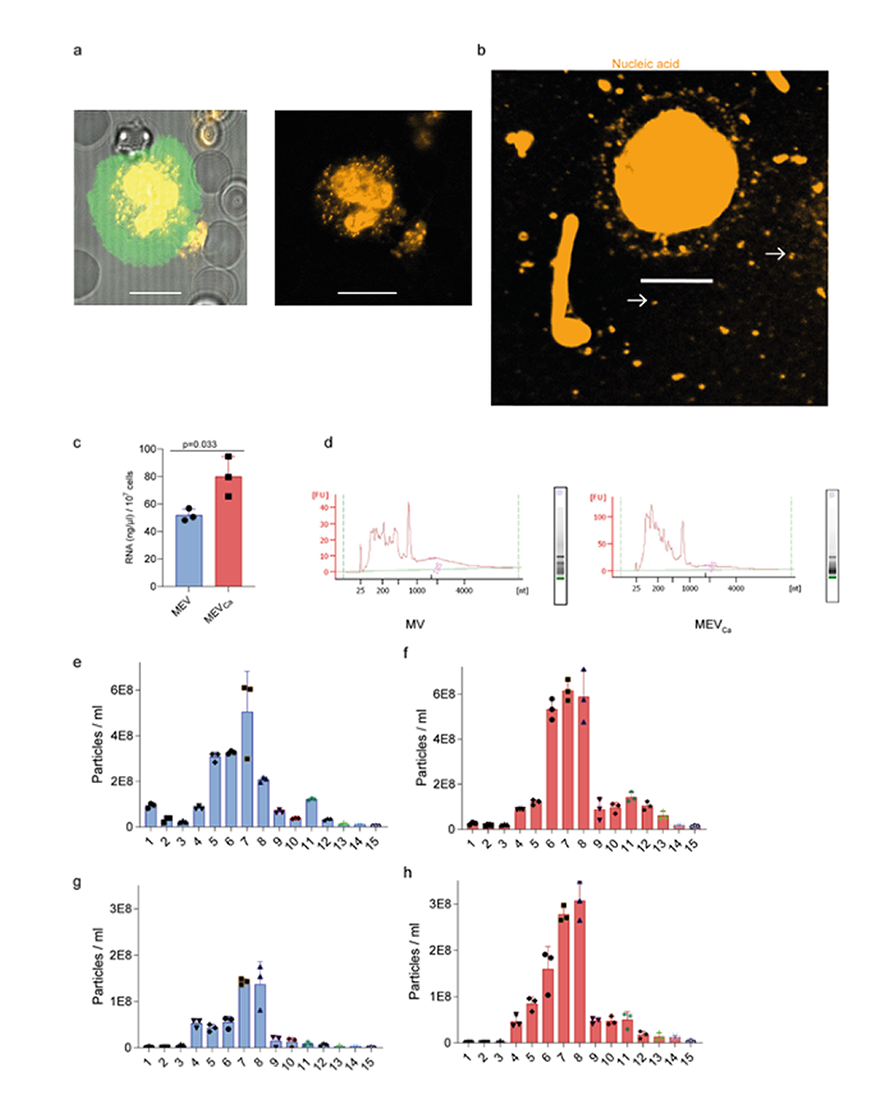

Supplement: FIG S1 [file mbio.03563-21-sf001.tif]

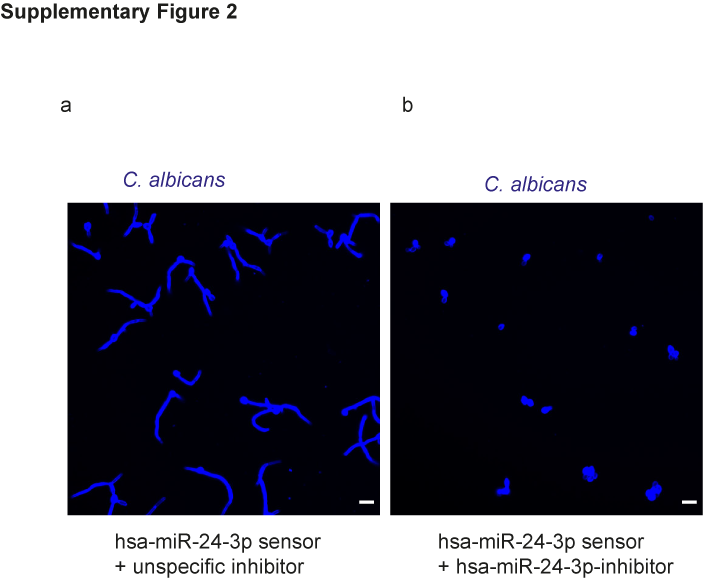

Supplement: FIG S2 [file mbio.03563-21-sf002.tif]

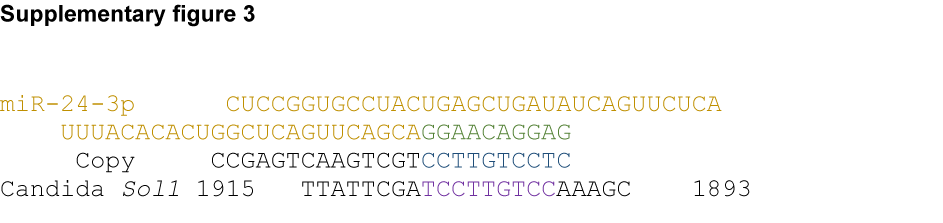

Supplement: FIG S3 [file mbio.03563-21-sf003.tif]
